# Pleiotropic Roles for the *Plasmodium berghei* RNA Binding Protein UIS12 in Transmission and Oocyst Maturation

**DOI:** 10.3389/fcimb.2021.624945

**Published:** 2021-03-05

**Authors:** Katja Müller, Olivier Silvie, Hans-Joachim Mollenkopf, Kai Matuschewski

**Affiliations:** ^1^ Department of Molecular Parasitology, Institute of Biology, Humboldt University, Berlin, Germany; ^2^ Parasitology Unit, Max Planck Institute for Infection Biology, Berlin, Germany; ^3^ Centre d’Immunologie et des Maladies Infectieuses, INSERM, CNRS, Sorbonne Université, Paris, France; ^4^ Core Facility Microarray/Genomics, Max Planck Institute for Infection Biology, Berlin, Germany

**Keywords:** malaria, *Plasmodium*, RNA binding protein (RBP), translational regulation, RNA, gametocyte, oocyst

## Abstract

Colonization of the mosquito host by *Plasmodium* parasites is achieved by sexually differentiated gametocytes. Gametocytogenesis, gamete formation and fertilization are tightly regulated processes, and translational repression is a major regulatory mechanism for stage conversion. Here, we present a characterization of a *Plasmodium berghei* RNA binding protein, UIS12, that contains two conserved eukaryotic RNA recognition motifs (RRM). Targeted gene deletion resulted in viable parasites that replicate normally during blood infection, but form fewer gametocytes. Upon transmission to *Anopheles stephensi* mosquitoes, both numbers and size of midgut-associated oocysts were reduced and their development stopped at an early time point. As a consequence, no salivary gland sporozoites were formed indicative of a complete life cycle arrest in the mosquito vector. Comparative transcript profiling in mutant and wild-type infected red blood cells revealed a decrease in transcript abundance of mRNAs coding for signature gamete-, ookinete-, and oocyst-specific proteins in *uis12(-)* parasites. Together, our findings indicate multiple roles for UIS12 in regulation of gene expression after blood infection in good agreement with the pleiotropic defects that terminate successful sporogony and onward transmission to a new vertebrate host.

## Introduction

During the complex life cycle of *Plasmodium*, malarial parasites switch between different host cells, extra- and intracellular life cycle forms, and an invertebrate and a vertebrate host. As the parasites progress in their developmental program, and particularly after switching host, adaptation to new environmental conditions, including temperature and immune defense, is central. For instance, *Plasmodium* gametocytes must remain infectious in the blood of the vertebrate host until they are eventually transmitted to the vector during a blood meal. The gametocytes need to quickly adapt to new environmental factors, leave the digestive environment of the blood meal, and colonize the mosquito midgut (Billker et al., 1998; Billker et al., 2004). To swiftly accomplish a host switch, transcription and translation are tightly coordinated.

Transcriptional reprogramming is controlled by the apicomplexan Apetala-2 (Api-AP2) transcription factors (Balaji et al., 2005; Painter et al., 2011). Many genes needed for gametocytogenesis and early events of mosquito colonization are transcribed by the interplay of the *Plasmodium* transcription factors AP2-G and AP2-I, while asexual gene expression is repressed by AP2-G2 and AP2-FG controls female-specific gene regulation (Sinha et al., 2014; Yuda et al., 2015; Josling et al., 2020; Yuda et al., 2020). This transcriptional switch leads to the expression of mRNAs which are required for the multiple steps from gametocytogenesis to zygote formation until the motile ookinete stage is reached and the next checkpoint in transcription control is initiated by the AP2-O 1-4 family (Kaneko et al., 2015; Modrzynska et al., 2017). Therefore, a tight fine-tuning is essential to synthesize tailored proteins at the right time.

Translational repression occurs during host switch to keep gametocytes and sporozoites in a latent state. Accordingly, translation of pre-synthesized mRNAs, which are required for mosquito colonization, occurs only after transmission and constitutes a mechanism to be prepared for the new host environment. In gametocytes a large number of genes have been described to be translationally repressed (Le Roch et al., 2004; Mair et al., 2006; Lasonder et al., 2008; Mair et al., 2010; Zhang et al., 2010; Lasonder et al., 2016). Translational repression is particularly dominant in female gametocytes and affects a wide range of mRNAs, including those that encode surface proteins, e.g., P25, P28, and CCP2, transcriptional regulators, e.g., AP2-O, and proteases, e.g., plasmepsin IV. A conserved U-rich 47 nucleotide-long cis-acting consensus motif in the 5’ and 3’ UTRs of a subset of translationally repressed transcripts in *P. berghei* gametocytes was shown to be important in translational regulation in gametocytes (Hall et al., 2005; Braks et al., 2008). In the female gametocyte, the DDX6-class DEAD box RNA-helicase DOZI (development of zygote impaired) is bound to its target transcripts in a large storage mRNP complex and silences their translation (Mair et al., 2006; Mair et al., 2010; Guerreiro et al., 2014). The Pumilio-family RNA binding protein Puf2 increases proliferation and decreases stage differentiation. As a consequence, deletion of *P. berghei* and *P. falciparum Puf2* resulted in higher gametocyte rates and premature transformation of sporozoites in the mosquito salivary glands (Miao et al., 2010; Gomes-Santos et al., 2011; Muller et al., 2011; Lindner et al., 2013). In *pfpuf2(-)* parasites, an up-regulation of many gametocyte specific transcripts, mostly independent of those that are regulated in *dozi(-)/cith(-)*,** was observed (Miao et al., 2010). Specific binding of *pfs25*, *pfs28*, and *Plasmepsin VI* mRNA by the *Pf*Puf2 protein was shown by RNA immunoprecipitation (Miao et al., 2013).

During host switch to the mosquito vector Puf2 negatively regulates gametocyte formation, and a post-transcriptional activator of gametocyte formation still remains elusive. In a complex with other proteins DOZI control of mRNA translation occurs much later, shortly after fertilization of female gametocytes inside the mosquito. Therefore, it is likely that additional factors participate in the regulation of post-transcriptional control of gene expression during mosquito transmission. In the present study we characterized an RNA binding protein, originally identified as up-regulated in infectious sporozoites gene 12 (*UIS12*), which is also highly expressed in gametocytes. A suppression subtractive hybridization screen aimed at discovering genes which are up-regulated in salivary gland sporozoites first described the *P. berghei* RNA binding protein UIS12 (PBANKA_0506200) (Matuschewski et al., 2002). As expected, protein prediction programs indicate an intracellular localization. Comparison of transcriptome and proteome data indicate that *P. berghei UIS12* is expressed in sporozoites, but the corresponding protein is not detectable, suggesting that UIS12 expression is regulated post-transcriptionally (Lasonder et al., 2008; Le Roch et al., 2004; Lindner et al., 2019). Due to the presence of the two RRM motifs, UIS12 is likely involved in post-transcriptional control of gene expression in gametocytes and pre-erythrocytic stages. Employing experimental genetics, the role of *UIS12* for parasite life cycle progression was analyzed with an emphasis on a potential function in post-transcriptional regulation.

## Results

### The RNA Binding Protein *Pb*UIS12 Is Expressed at Multiple Life Cycle Stages

**Figure 1:**
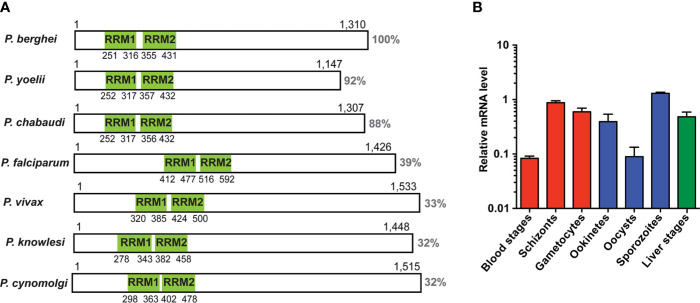
Expression analysis of UIS12. **(A)** Schematic display of protein primary structure illustrating length, homology and position of the two predicted RRM domains (green) of the UIS12 orthologues of different *Plasmodium* species. Protein sequence identity is shown to the right. Gene identification and accession numbers are as follows: *P. berghei*, PBANKA_0506200; *P. yoelii*, PY17X_0507300; *P. chabaudi*, PCHAS_0506300; *P. falciparum*, PF3D7_0823200; *P. vivax*, PVX_001965; *P. knowlesi*, PKNH_0606300; *P. cynomolgi*, PCYB_061630. **(B)** Expression profile of *UIS12* in different *P. berghei* life cycle stages; mixed blood stages, schizonts, gametocytes (red), ookinetes, oocysts, sporozoites (blue), and 24 h liver stages (green). Expression levels were quantified by qRT-PCR, and data were normalized to GFP, constitutively expressed under the *PbEF1α* promoter (cl507) (Janse et al., 2006a). Mean values ( ± SD) from two independent experiments are shown.

**Figure 2:**
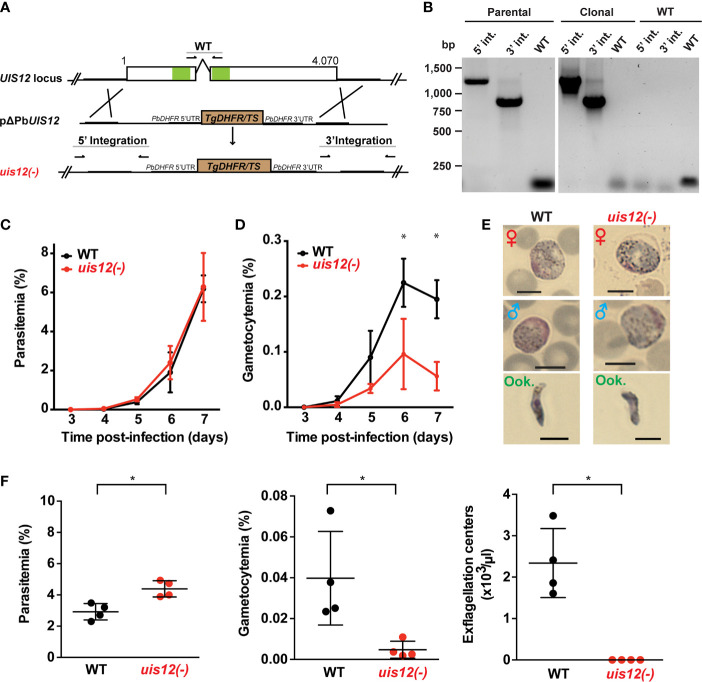
Normal asexual blood stage growth in *uis12(-)* parasites, but reduced gametocyte development and exflagellation. **(A)** Graphical scheme showing the replacement strategy to generate *uis12(-)* parasites, and *UIS12* gene structure. The *UIS12* locus was replaced by double-homologous recombination with the linearized replacement plasmid pΔPBANKA_UIS12, which includes a resistance marker (*Toxoplasma gondii DHFR/TS*) and the 5’ and 3’ flanking regions of *UIS12*. Arrows and lines indicate positions of knockout- and wild-type-specific oligonucleotide positions and PCR fragments, respectively. The RNA recognition motif domains (RRM) are shown in green. **(B)** Confirmation of *UIS12* gene disruption by diagnostic PCR. Genomic DNA of parental *uis12(-)*, clonal *uis12(-)* clone 2, and wild-type parasites served as templates. 5’- and 3’-integration-specific primer combinations amplify the predicted fragment only in the recombinant locus. Wild-type-specific primer pairs that do not produce a PCR fragment in the recombinant locus amplified the wild-type locus (right panel). Absence of residual wild-type confirms a clonal *uis12(-)* line (central panel). Time courses of parasitemia **(C)** and gametocytemia **(D)** of *uis12(-)* (clone 1, red) and wild-type (WT) (black) parasites, starting three days after intravenous injection of 10,000 mixed blood stages into C57BL/6 mice (*n*=5). Daily microscopic analysis of Giemsa-stained blood films was used to determine parasitemia and gametocytemia. **(C)** Parasitemia, percentage of asexual parasites per total erythrocytes **(D)** gametocytemia, percentage of gametocytes per total erythrocytes. Means of parasitemia and gametocytemia ( ± SD) are shown. *p < 0.05 (multiple t-tests, one per row). **(E)** Gametocytes and ookinetes of *uis12(-)* (clone 1) are morphologically indistinguishable from wild-type (WT). Shown are microscopic images of Giemsa-stained female (♀) and male (♂) gametocytes and ookinetes (Ook., from ookinete cultures). Brightfield microscopy, magnification 1,000-fold. Scale bars, 5 µm. **(F)** Asexual and sexual blood stage development. Infected blood (C57BL/6 mice; *n*=4) was analyzed four days after injection of 10^7^ *uis12(-)* (clone 1, red) or WT (black)-infected erythrocytes, respectively. Plots show parasitemia (left), gametocytemia (center), and numbers of exflagellation centers per microliter of mouse tail blood (right). Results are expressed as means ( ± SD). *p < 0.05 (Mann-Whitney test).

We initiated our analysis by an NCBI conserved domain database (CDD) search (Marchler-Bauer et al., 2015), which revealed two ~70 amino acid long RNA-recognition motifs (RRM), belonging to the class of RNA-binding domains (RBD) (Dreyfuss et al., 1988), in the *P. berghei* UIS12 protein sequence. The *UIS12* gene, and particularly the two RRM domains, are highly conserved between different *Plasmodium* UIS12 orthologs (**Figure 1A**), while in a candidate *Toxoplasma gondii* ortholog (TGME49_268380) the conservation is limited to the two RRM domains only. In general, the amino- and carboxy-terminal regions are less conserved, and all *Plasmodium* ortholog proteins have similar sizes of ~1,300 amino acid residues. The *PbUIS12* coding region has a total length of 4,070 base pairs and consists of two exons (**Figure 2A**). Of note, the two regions encoding the RRM domains are separated by the single intron, which also exhibits a degree of conservation.

We next measured *UIS12* transcript abundance throughout the *P. berghei* life cycle by quantitative RT-PCR (**Figure 1B**). In good agreement with the previous notion of up-regulation in salivary gland sporozoites, *UIS12* mRNA steady state levels are low in midgut oocysts and highest in salivary gland sporozoites. High *UIS12* transcript levels were also detected in early (24h) liver stages. In mixed asexual blood stages *UIS12* mRNA levels are low, whereas in schizonts, gametocytes and ookinetes, considerable expression was detected. Our observations mostly corroborate published data (Howick et al., 2019; Otto et al., 2014). However, we did not specifically analyze ring stages or distinguished between male and female gametocytes (Yeoh et al., 2017). Together, *UIS12* is expressed at multiple points of the *P. berghei* life cycle, indicative of multiple functions during life cycle progression and parasite stage conversion.

### Targeted Gene Disruption of the RNA-Binding Protein *PbUIS12*

In order to study the gene function, we generated a *UIS12* loss-of-function mutant. *PbUIS12* deletion was generated by double homologous recombination employing a replacement strategy, as described previously (van Dijk et al., 1995) (**Figure 2A**).

The *UIS12* targeting construct (pΔPBANKA_UIS12) comprised ~500 bp each of the 5’ and 3’ non-coding sequences flanking a *TgDHFR*/*TS* pyrimethamine resistance cassette for positive selection with the anti-malarial drug pyrimethamine. Upon a double homologous recombination event, the *UIS12* gene is expected to be replaced by the selectable marker (**Figure 2A**). As recipient parasites we used *P. berghei* (strain: ANKA; clone: cl507), which expresses the fluorescent protein GFP under the control of the constitutive *eIF1α* promoter (Janse et al., 2006a). After transfection we recovered parental *uis12(-)* blood stage populations, from which clonal lines were generated by injection of limiting serial dilutions into mice. The desired recombination event was confirmed by diagnostic PCR, which confirmed the presence of 5’ and 3’ integration in the parental and the clonal *uis12(-)* populations, and by the absence of wild-type-specific PCR fragments in the clonal *uis12(-)* populations. During the course of our *UIS12* knock-out analysis and to further corroborate our findings we successfully generated an independent second *uis12(-)* knockout line (*uis12(-) clone 2*), in a different *P. berghei* ANKA recipient line, Bergreen, which constitutively expresses GFP under the control of the *HSP70* promoter (Kooij et al., 2012) (**Figure 2B**: clone 2; **Figure S1A**: clone 1).

At this stage, we could already conclude that the successful generation of clonal *uis12(-)*-knockout lines demonstrates that the gene is not essential for normal progression of asexual blood stages, although *UIS12* is expressed considerably in schizonts (**Figure 1B**).

### Impaired Gametocyte Development of *uis12(-)* Parasites

After successful selection of *uis12(-)* parasites, we first examined asexual and sexual blood stage growth. To this end, C57BL/6 mice (*n*=5) were infected by intravenous injection of either 10,000 *uis12(-)* or WT-infected erythrocytes. Starting three days after infection the mice were monitored by daily microscopic examination of thin blood films, and parasitemia and gametocytemia were quantified (**Figures 2C, D**). Parasitemia increased daily in wild-type- and *uis12(-)*-infected mice to a similar extent. On day 7, wild-type and *uis12(-)*-infected mice started developing symptoms indicative of onset of experimental cerebral malaria, supporting the notion that *uis12(-)* parasites display virulence typically seen in infections with *P. berghei* ANKA parasites.

The blood films were also analyzed for the presence of male and female gametocytes (**Figure 2D**). This analysis revealed that, in contrast to normal asexual blood infection, gametocytemia was significantly reduced in *uis12(-)* parasites as compared to the wild-type. *Uis12(-)* gametocyte numbers were already slightly reduced on day four after infection, when the first fully mature gametocytes became visible. The difference between WT and *uis12(-)* parasites increased further over time. Examination of size and morphology of male or female gametocytes and ookinetes revealed no difference between the two parasite lines (**Figure 2E**). The presence of ookinetes indicates that *uis12(-)* gametocytes are able to fertilize and subsequently form a zygote that develops into an ookinete. Both, male and female gametocytes were found in the expected female biased proportions. Together, the data indicate that UIS12 is dispensable for asexual growth, but plays an important role during gametocytogenesis. Analysis of the second *uis12(-)* clone corroborated these findings (**Figures S1B, C**).

### Exflagellation Is Markedly Reduced in *uis12(-)* Parasites

The potential of male gametocytes to produce motile microgametes is an active process and termed exflagellation. In both *uis12(-)* clonal lines, exflagellation events were detected very rarely by microscopic examination of undiluted blood from *uis12(-)* infected mice at any given time point. Yet, this already showed that *uis12(-)* male gametocytes are able to produce motile male gametes, albeit at very low frequencies. To quantify the exflagellation centers, C57BL/6 mice (*n*=4) were injected intravenously with a high dose of 10^7^ blood stages. Four days later, parasitemia, gametocytemia and the number of exflagellation centers were enumerated (**Figure 2F**, *uis12(-)* clone 1). Interestingly, in this experiment, the parasitemia was slightly, but significantly higher in *uis12(-)*-infected mice. In agreement with our first experiment (**Figure 2D**), gametocytemia was 8.6-fold reduced in *uis12(-)* parasite-infected mice. Exflagellation was not visible under these conditions, i.e., 1:25 diluted blood, in *uis12(-)*-infected mice (**Figure 2F**). Even after an extended incubation or when examined the following days, exflagellation was not detectable. In conclusion, male *uis12(-)* gametocytes retain the ability to progress to functional microgametes, but at a very low rate. The defect in exflagellation is more severe than the reduction in gametocytemia indicating additive defects in absence of *UIS12* upon progression from blood infection to transmission to the insect vector.

### Reduced Oocyst Formation By *uis12(-)* Gametocytes, But Failure to Produce Sporozoites

**Figure 3:**
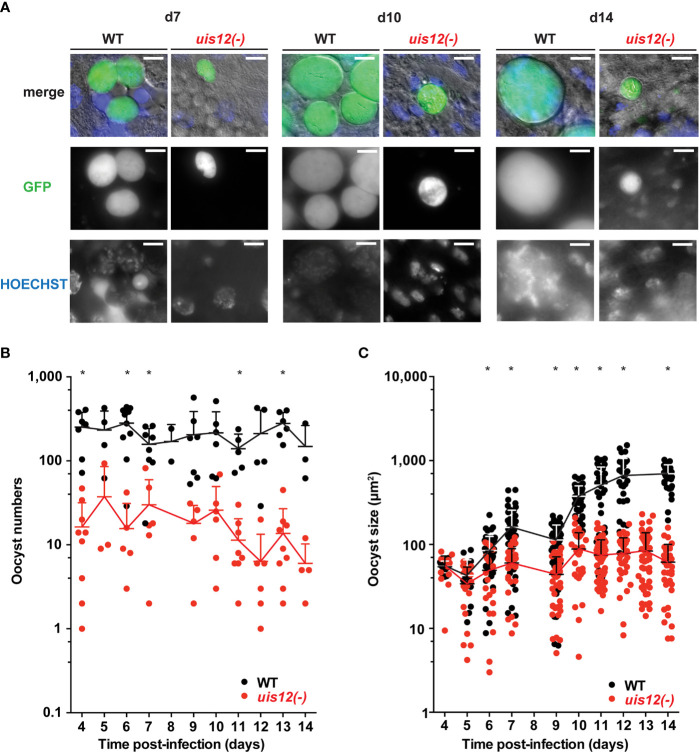
Disruption of *UIS12* leads to a reduced oocyst size and a defect in sporozoite formation. **(A)** Shown are representative live micrographs of *uis12(-)* and wild-type oocysts 7, 10 and 14 days after an infectious blood meal. GFP (green) is constitutively expressed under the *HSP70* promoter (*P. berghei* ANKA Bergreen) (Kooij et al., 2012). *Uis12(-)* clone 2 was used for this experiment. Nuclei were stained with Hoechst (blue). Note the reduced density and size of *uis12(-)* oocysts, and absence of sporozoites in *uis12(-)* oocysts. Magnification, 630-fold; scale bars, 10 µm. **(B, C)** Time course of oocyst numbers per infected mosquito midgut **(B)** and oocyst size (µm^2^) **(C)** at different time points after midgut infection (*uis12(-)* clone 2, red; WT, black). *p < 0.05 (multiple t-tests).

The severe defect in *in vitro* exflagellation implied a large impact in mosquito colonization. However, after transmission to *Anopheles stephensi* mosquitos, *uis12(-)* parasites were able to establish midgut infections and produce oocysts, showing that early events of midgut colonization are not abolished *in vivo* (**Figure 3**). The mean mosquito infection prevalences were 54% ( ± 18%) for wild-type (*n*=5) and 46% ( ± 23%) for *uis12(-)* (*n*=5).

This finding prompted us to quantify oocyst maturation. To this end, midguts were isolated daily starting on day 4 after mosquito infection up until day 14. Oocyst numbers and morphology as well as their ability to produce sporozoites were analyzed by fluorescence microscopy (**Figure 3**, *uis12(-)* clone 2). In *uis12(-)-*infected mosquitoes, the number of oocysts per midgut was more than 10-fold reduced as compared to wild-type, consistent with reduced gametocyte numbers and the very low exflagellation rate (**Figure 3B**). Strikingly, in the absence of *UIS12* oocysts were markedly smaller in size (**Figures 3A, C**). While up until day 4 the oocyst size was comparable between both groups, over time *uis12(-)* oocysts displayed an abnormal growth pattern. Their size remained unchanged, whereas the WT oocyst diameter increased ~10-fold over the following ten days. This finding indicates that the early events of oocyst development, including transformation of the motile ookinete to the spherical oocyst, and the initial increase in biomass (Carter et al., 2007) are reduced, but not completely abolished. An additional defect leads to arrested oocyst maturation. The analysis of the second *uis12(-)* clone corroborated these findings (**Figures S1D–F**).

Upon close examination at later time points, *uis12(-)* oocysts displayed little or no nuclear staining, harbored many large vacuoles, and frequently were surrounded by an apparently wrinkled oocyst wall (**Figure 3A**, **Figure S1F**). As a consequence, no sporozoites were seen inside *uis12(-)* oocysts. This lack of sporozoite formation was confirmed by analysis of extracted midguts; no midgut- associated sporozoites were detectable. In WT-infected midguts sporoblast formation was observed inside the oocysts starting around day 11 after infection, and from day 12 onwards oocysts started to fill with sporozoites (Matuschewski, 2006) (**Figure 3A**). In contrast, *uis12(-)* midguts remained free of sporozoites even until late time points of mosquito infection, excluding delayed sporozoite formation.

In good agreement, initial microscopic screening of salivary gland extracts of *uis12(-)*-infected mosquitoes for presence of sporozoites did not return a single sporozoite. To obtain independent support for the complete absence of sporozoites in *uis12(-)*-infected mosquitoes, we inoculated C57BL/6 mice with salivary gland extracts of 20 *uis12(-)*-infected mosquitoes. In addition, we exposed C57BL/6 mice to ~100 *uis12(-)*-infected mosquitoes (**Table 1**, both clones). In sharp contrast to control infections with wild-type-infected mosquitoes under similar conditions, none of the attempts to transmit *uis12(-)* parasites resulted in blood stage infections (**Table 1**). This defect in transmission confirms that *uis12(-)* parasites are unable to complete their development inside the mosquito.

**Table 1:** No malaria transmission by *uis12(-)*-infected mosquitoes.

Parasites	Route of injection^a^	Infected/Injected	Pre-patency (days)^b^
WT 507cl	10,000 sporozoites i.v.	5/5	3
*uis12(-)* clone 1	mosquito bite (~100 mosquitoes)	0/5	N.A.
	salivary gland extract i.v. (20 mosquitoes)	0/4	N.A.
WT Bergreen	mosquito bite (~100 mosquitoes)	3/3	3
*uis12(-)* clone 2	mosquito bite (~100 mosquitoes)	0/3	N.A.
	salivary gland extract i.v. (20 mosquitoes)	0/3	N.A.

a C57BL/6 mice were exposed to the bites of ~100 infected mosquitoes for 15–20 min or injected intravenously (i.v.) with salivary gland extract isolated from 20 mosquitoes (uis12(-)) and, as a control, 10,000 sporozoites (WT). Data are pooled from two separate experiments.

b the pre-patent period represents the days until detection of the first infected erythrocytes by microscopic thin blood film examination after sporozoite inoculation. N. A. not applicable. The colors were used to highlight different conditions.

### Deregulation of Transcripts Important for Mosquito Transmission in *uis12(-)* Blood Stages

We hypothesized that due to the presence of the two RRM motifs, UIS12 is likely involved in post-transcriptional control of gene expression. Reduced gametocyte production, exflagellation, and oocyst development detected in *uis12(-)* parasites suggests that UIS12 might be regulating target mRNAs and alter expression of the respective proteins, which in turn play important roles in these stages.

**Figure 4:**
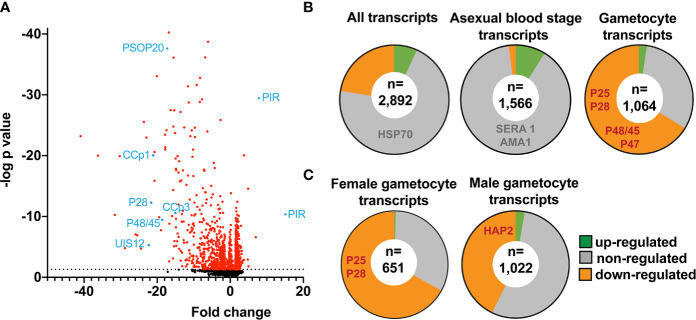
Down-regulation of distinct mRNAs coding for signature gamete-, ookinete- and oocyst-specific proteins in *uis12(-)* blood stage parasites. Shown are mean values of microarray results of total RNA isolated from mixed infected erythrocytes from *uis12(-)* (clone 1)*-* and WT- infected mice from two biological replicates. **(A)** Shown is a volcano-plot illustrating the fold change of the expression levels and the negative log *p*-values of all analyzed 2,890 *P. berghei* genes. The dotted black line represents a *p*-value of 0.05 and all transcripts with a *p*-value <0.05 are shown in red. Exemplary transcripts are highlighted and labeled in blue. **(B)** Pie charts displaying the proportions of up- (green >2), non- (grey), and down- (orange <-2) regulated transcripts among all transcripts (upper left), blood stage-specific transcripts (center), and gametocyte-specific transcripts (upper right) (Otto et al., 2014). Exemplary transcripts are listed in the respective region of the chart. The number of transcripts analyzed is shown in a white circle inside the center. **(C)** Pie charts displaying the proportions of up- (green >2), non- (grey), and down- (orange <-2) regulated transcripts among female (left) and male (right) gametocyte-specific transcripts (Yeoh et al., 2017). The number of transcripts analyzed is shown in a white circle inside the center.

To profile the transcriptome of *uis12(-)* parasites, we performed a comparative microarray analysis on *uis12(-)* and wild-type mixed blood stage RNAs (**Figure 4**, **Figure S2**). Since the first defect is detected during gametocyte formation, we limited our analysis to mixed blood stages, that include the asexual blood stages, from which gametocytes originate. Attempts to retrieve sufficient *uis12(-)* gametocytes for a microarray failed. The microarray analysis was performed with two biological replicates (R1 and R2), both with *uis12(-)* clone 1. For both replicates, parasitemia of the samples were comparable (R1: WT 6.2%; *uis12(-)* 6.3%; R2: WT 6.4%; *uis12(-)* 8.7%). As expected, gametocytemia was reduced in *uis12(-)* parasites (R1: WT 0.2%; *uis12(-)* 0.06%). Each microarray experiment was performed by dual-color hybridizations, and a color-swap dye-reversal was included in order to compensate for dye-specific effects. In this microarray analysis, the -fold change of the expression levels of 2,890 *P. berghei* genes could be successfully compared (**Figure 4A**, **Figures S2A, D**).

Overall, absence of *PbUIS12* correlated with a global perturbation of 30% of all mRNA expression levels (threshold, <-2 or >2). More transcripts were down-regulated (646, 23%) than up-regulated (199, 7%). The mean down-regulation was -6.4-fold, whereas the mean up-regulation was +2.5-fold (GEO Series accession number: GSE152686). As expected, the *UIS12*-knockout was independently confirmed by the microarray analysis; *UIS12* transcripts were down-regulated by -22.3-fold (**Table 2**). Within the 25 most down-regulated transcripts known proteins required for the switch to the mosquito vector, CCp1 (LAP2), LAP5, P48/45, and P28, as well as numerous previously unrecognized proteins are represented (**Table 2**). The list of the 25 most up-regulated genes contains, among others, PIR proteins (**Table S1**). Remarkably, many transcripts that were down-regulated more than 10-fold in *uis12(-)* blood stages, are reported to be expressed in gametocytes (Hall et al., 2005). We also analyzed the proportions of up-, down-, and non-regulated transcripts and found distinctions between stage-specific RNAs, as defined by RNA sequencing data (Otto et al., 2014). For instance, gametocyte transcripts were defined by >2-fold up-regulation in gametocytes as compared to asexual blood stages (**Figure 4B**, **Table 3**, **Figures S2B, E**). In good agreement with normal replication rates, very few asexual blood stage-specific transcripts were down-regulated in *uis12(-)* parasites. Instead, the proportion of up-regulated transcripts was highest for asexual blood stage-specific mRNAs, which might reflect over-representation of these stages due to defects in gametocytogenesis. However, signature transcripts, like *AMA1* (-0.3), *HSP70* (-1.6), *MSP1* (1.7) and *MSP8* (1.5), were neither up- nor down-regulated in the absence of *UIS12* (**Figure 4B**, **Table 3**). We also noted that the mRNA levels of many signature mosquito stage-specific proteins were down-regulated in *uis12(-)* mixed blood stages. In addition, we also compared the proportions of up-, down- and non-regulated transcripts and found distinctions between male- and female-specific transcripts, as defined by RNA sequencing data (Yeoh et al., 2017) (**Figure 4C**, **Figures S2C, F**). While 67% of the female gametocyte transcripts were down-regulated in absence of *UIS12,* only 43% of male-specific transcripts were down-regulated, indicating differential impact of UIS12 on male and female gametocyte-specific transcripts in mixed blood stages.

**Table 2:** The top 25 down-regulated genes in blood stages of *uis12(-) vs.* wild-type.

Gene ID	Product description	Mean -fold change R1^a^ R2^b^	-fold change R1^a^	-fold change R2^b^
PBANKA_0806800	unknown	-41.0	-37.5	-44.4
PBANKA_1449000	candidate microgamete surface protein MiGS	-36.2	-25.4	-47.0
PBANKA_1349400	candidate DNA replication complex GINS protein	-31.6	-29.0	-34.2
PBANKA_1340400	candidate lactate dehydrogenase	-30.2	-24.2	-36.3
PBANKA_1400400	fam-b-protein	-28.8	-14.3	-43.4
PBANKA_1361600	E1-E2 ATPase	-25.9	-25.9	-25.9
PBANKA_1040300	fam-b-protein	-25.4	-18.5	-32.3
PBANKA_1315300	LCCL domain-containing protein (LAP5)	-24.4	-27.4	-21.4
PBANKA_0413000	unknown	-23.6	-29.3	-18.0
PBANKA_1246600	cleft-like protein 1	-22.9	-9.7	-36.2
PBANKA_0506200	RNA binding protein (UIS12)	-22.3	-31.7	-12.8
PBANKA_0514900	ookinete surface protein P28	-21.6	-15.5	-27.8
PBANKA_1300700	LCCL domain-containing protein (CCp1)	-21.1	-20.7	-21.4
PBANKA_0719700	unknown	-20.7	-19.2	-22.2
PBANKA_0707100	inner membrane complex protein 1i	-20.7	-26.5	-15.0
PBANKA_1334900	unknown	-20.4	-20.1	-20.7
PBANKA_1353800	unknown	-20.1	-26.1	-14.1
PBANKA_1432400	perforin-like protein 2	-19.4	-16.9	-21.9
PBANKA_0608600	candidate EGF-domain protein	-19.0	-23.9	-14.0
PBANKA_1400900	unknown	-18.9	-20.3	-17.5
PBANKA_1359600	6-cysteine protein (P48/45)	-18.6	-21.0	-16.2
PBANKA_1106000	unknown	-17.9	-22.2	-13.5
PBANKA_1414800	unknown	-17.9	-19.6	-16.1
PBANKA_1120400	candidate inner membrane complex protein 1j	-17.8	-18.9	-16.7
PBANKA_1318600	unknown	-17.8	-15.9	-19.7

a biological replicate 1.

b biological replicate 2. The colors were used to highlight different conditions.

**Table 3:** Examples for deregulation of signature vector-stage transcripts in *uis12(-)* parasites.

	Known blood stage transcripts		Known transmission stage transcripts	
Up-regulated	Pb235 2.8			
Unaffected	AMA1 MSP8 HSP70 SERA3 MSP1	-0.3 1.5 -1.6 1.7 1.7	AP-2 G* p230p NPT1* Puf2	-0.9 -0.3 -1.3 -1.9
Down-regulated (< 10-fold)			CITH* Actin II CDPK4 MAPK 2 GAP45 AP2-0 CDPK3 Plasmepsin VI HAP2 MDV1	-2.3 -2.8 -3.1 -3.3 -5.1 -5.7 -5.8 -6.3 -7.5 -10
Down-regulated (> 10-fold)			CCp5* CCp4* CCp3* P47 CCp2* P25 P48/45 CCp1* P28	-11.2 -12.2 -15 -15 -15.3 -17.1 -18.6 -21.1 -21.6

Shown are exemplary blood and transmission stage transcripts. The mean -fold change represents steady-state mRNA levels in uis12(-) parasites compared to the corresponding WT parasite stages. *genes with a knock-out phenotype similar to uis12(-).

**Figure 5:**
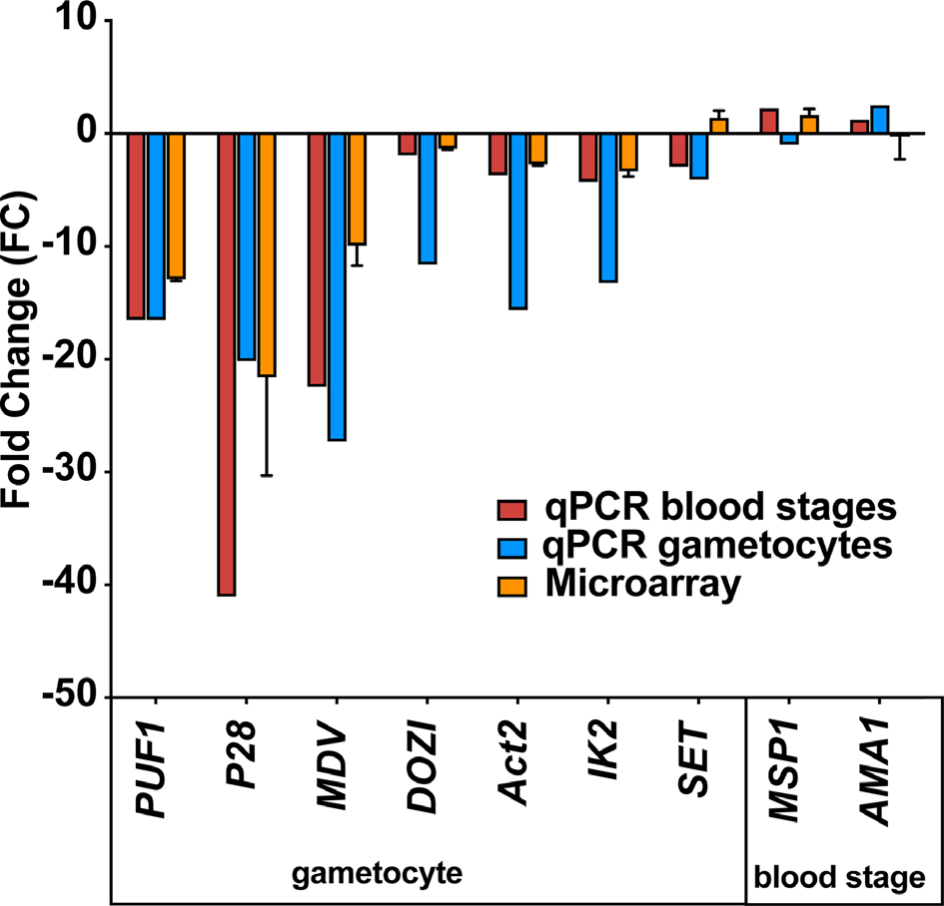
Confirmation of down-regulation of selected mRNAs by quantitative RT-PCR. Quantitative RT-PCR on selected blood stage and gametocyte transcripts validate the results of the microarray analysis. Shown are -fold changes of steady state mRNA levels of *uis12(-)* clone 1 compared to wild-type mixed blood stages for selected genes (*Puf1*, *P28*, *MDV*, *DOZI*, *Actin II*, *UIS1/IK2*, *SET*, *MSP1*, and *AMA1*). qRT-PCR data were normalized to the steady state levels of *HSP70* mRNA. The microarray data represent mean values of biological replicate 1 and 2 ( ± SD).

Next, we wanted to independently confirm the microarray results by quantitative RT-PCR of selected transcripts on blood stage and gametocyte mRNA from *uis12(-)* and WT samples (**Figure 5**). This analysis revealed a good agreement with the mean -fold changes determined in the microarray for gametocyte- and blood stage- specific transcripts. The gametocyte-expressed transcripts *Puf1*, *DOZI*, *UIS1/IK2*, *SET*, and *Actin II* (Mair et al., 2006; Pace et al., 2006; Deligianni et al., 2011; Gomes-Santos et al., 2011; Muller et al., 2011) showed similar -fold changes in both assays. Similarly, the blood stage-expressed transcripts *AMA1* and *MSP1* remained un-regulated. For some transcripts, e.g., male development factor (*MDV1*) (Lal et al., 2009), the reduction in *uis12(-)* parasites was more pronounced by quantitative RT-PCR in comparison to the microarray data. qRT-PCR on gametocyte mRNA samples were consistent with the data from asexual blood stages (**Figure 5**, *uis12(-)* clone 1). Overall, we could confirm the results of the microarray on these samples, indicating that the down-regulation of the gametocyte transcripts is a direct result of a specific UIS12-dependent regulation and not merely a consequence of the absence of gametocytes in *uis12(-)* mixed blood stages.

### Gene Ontology Analysis of the Deregulated Transcripts in *uis12(-)* Parasites

We next performed a gene ontology (GO) enrichment analysis (Ashburner et al., 2000) (**Tables S2** and **S3**). According to this analysis the 199 up-regulated and the 646 down-regulated transcripts are involved in 21 and 42 different biological processes, as described by distinct GO terms, respectively. Examples for GO terms of the set of down-regulated genes include reproduction, cell communication and phosphorylation (**Table S2**), in good agreement with the known roles of kinases in gametogenesis (Invergo et al., 2017). Examples for GO terms of the set of up-regulated genes include RNA processing and histone acetylation (**Table S3**), further indicative of perturbations in gene expression and mRNA translation in absence of *UIS12*.

### A 10-Nucleotide Motif Shared By Up-Regulated Transcripts in *uis12(-)* Parasites

**Figure 6:**
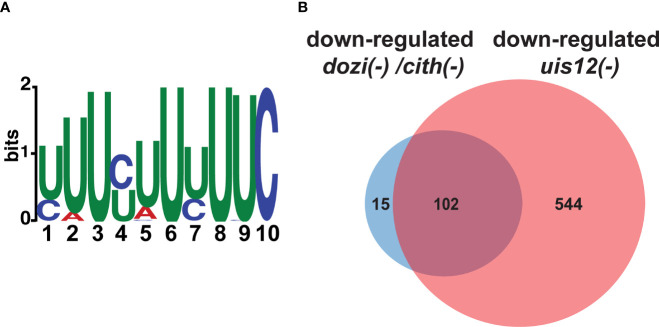
A motif is shared by up-regulated transcripts in *uis12(-)* parasites, and the comparison of *dozi(-), cith(-)* and *uis12(-)* target transcripts reveals possible overlapping functions. **(A)** Shown is a graphic display of the 10-nucleotide U-rich motif found in the ORF of transcripts that are up-regulated in absence of *UIS12* in the microarray with biological replicate 1. The size of the depicted nucleotide represents its probability at the respective position. **(B)** Venn diagram comparing shared down-regulated transcripts of the gametocyte microarray analyses performed for *dozi(-)* and *cith(-)* with a -fold change lower than -1 (Mair et al., 2010) and down-regulated transcripts in mixed blood stages of *uis12(-)* with a mean -fold change lower than -2.

Since UIS12 contains two RNA recognition motifs and the combination of at least two RRM domains allows the continuous recognition of a 8-10 nucleotide-long sequence motif with high affinity (Maris et al., 2005), we screened all ORFs, and 1,000 base pairs of the flanking 5’ UTR and 3’ UTR sequences of genes up-regulated >2.5-fold in replicate 1 for common 8-10 nucleotide-long motifs using MEME (Bailey et al., 2009) using the respective portions of the non-regulated transcripts as reference. While this search did not return a motif in either the 5’ or 3’ UTR sequences, we found a 10 nucleotide-long motif (TTTYTTTTTC) in the ORF (**Figure 6A**). This signature was found 751 times in 174 of the 220 up-regulated sequences (e-value: 4.2x10^-4^). We restricted our search to up-regulated transcripts, since down-regulation can at least be partially attributed to fewer gametocytes in mixed *uis12(-)* blood stages.

## Discussion

In this study, deletion of the candidate RNA binding protein UIS12 led to the discovery of a potential post-transcriptional regulator of gametocytogenesis and mosquito transmission. Our results underscore the key roles of a fine-tuned transcriptional control and post-transcriptional regulation during transmission of the malaria parasite between hosts. When *UIS12* is absent, fewer gametocytes are produced and *in vitro* exflagellation is severely impaired, yet not abolished. The defects of *uis12(-)* parasites during host switching cannot be compensated for in subsequent steps in the *Plasmodium* life cycle, and *uis12(-)* infection leads to fewer oocysts, which fail to undergo oocyst maturation. We can correlate these defects with a set of signature gametocyte and mosquito-stage transcripts that are down-regulated in *uis12(-)* parasites already during blood infection. Parasites lacking *UIS12* produce ~80% fewer gametocytes. Whether this reduction is due to a defect in sexual conversion or later during the maturation and/or survival of the sexual stages cannot be distinguished in the *P. berghei in vivo* model, where gametocytes are constantly formed. Both male and female gametocytes account for this reduction, indicative of a role of *UIS12* in early events of gametocytogenesis, perhaps in conjunction with transcriptional control by AP2-G, AP2-I, AP2-FG, and AP2-G2. Gene deletion of *AP2-G* resulted in a complete loss of gametocytes and the deletion of *AP2-G2* in a major reduction of gametocytes (Sinha et al., 2014). While *AP2-G* expression was unaffected in *uis12(-)* parasites, *AP2-G2* was not included in our microarray screen.

*Plasmodium falciparum* possesses at least 189 candidate RNA-binding proteins (RBP), which comprise 3.5% of all annotated genes. In good agreement with the complex regulation network required for stage conversion, a third of all RBPs show elevated expression during the gametocyte stage (Reddy et al., 2015). In contrast to a broad role in gene regulation, such as translational inhibition by phosphorylation of eIF2α, RRM-containing RBPs are mainly known for their tight RNA-specific control of RNA processing, export, and stability. The presence of tandem RRM domains, a feature observed in UIS12, ensures high sequence specificity (Maris et al., 2005).

Whether UIS12 is a positive regulator of gametocytogenesis and how it influences this process awaits further molecular and biochemical analysis, including RNA-pull down studies to capture UIS12 target transcripts. The role of UIS12 in promoting gametocytogenesis shares similarities with another RNA-binding protein, the pumilio-familiy RNA-binding protein *Puf1* which was shown to function in *P. falciparum* gametocyte maturation, and especially of female gametocytes (Shrestha et al., 2016). Both proteins likely inhibit asexual propagation and, thereby, promote gametocyte formation. A related pumilio-familiy RNA-binding protein, *Puf2*, exerts the corresponding function in sporozoites and prepares them for transmission to the vertebrate host (Miao et al., 2010; Gomes-Santos et al., 2011; Muller et al., 2011). We note that UIS12 likely executes a second function, similar to the one analyzed in the present study, in pre-erythrocytic stages, the stage where it was originally identified (Matuschewski et al., 2002), but the complete block in sporogony prevented us from studying this by classical reverse genetics. It, thus, still remains unclear if *UIS12* also plays a role in the host switch back to the mammalian host. Of note, in sporozoites translational repression of *UIS12* was described, and the protein is only detectable in liver stages (Lindner et al., 2019). Accordingly, a stage-specific knockout, which depletes *UIS12* during sporogony is required to study its cellular roles for sporozoite transmission and liver infection.

The few remaining *uis12(-)* gametocytes were able to form male and female gametes, fertilize, and form ookinetes. Accordingly, numbers of oocysts are extremely low in *uis12(-)-*infected *Anopheles* mosquitoes. Complete life cycle arrest in *uis12(-)* infections occurs at an early time-point after oocysts are formed, indicating that the events leading to oocyst formation are not impaired (Carter et al., 2007).

Our microarray analysis revealed a sharp drop in the steady state mRNA levels of numerous well-characterized *Plasmodium* genes. The *P. berghei* LCCL protein family members CCp1 and lectin adhesive-like protein 5 (LAP5) are expressed in female gametocytes and ookinetes. *Pb*LAP proteins are important for sporogenesis inside oocysts, yet known to be expressed already in gametocytes (Pradel et al., 2004; Trueman et al., 2004; Raine et al., 2007; Lavazec et al., 2009; Saeed et al., 2010). The surface proteins of the 6-cysteine repeat protein family P48/45 are essential for male gametocyte fertility (van Dijk et al., 2001). P25 and P28 are surface proteins of sexual stages, with distinct roles in ookinete to oocyst transformation (Siden-Kiamos et al., 2000; Tomas et al., 2001). Interestingly, some transcripts that are severely depleted, including P28, AP2-O, CCP2, and CCP4, have been reported to be translationally controlled (Vervenne et al., 1994; Saeed et al., 2013; Lasonder et al., 2016). Strikingly, the function of some of these repressed targets extends far beyond the ookinete stage, and the corresponding proteins are important for oocyst and sporozoite formation, illustrating the complexity of regulation of post-transcriptional control of gene expression in gametocytes (Lasonder et al., 2016). A deregulation of mRNAs already in mixed blood stages in absence of *UIS12* might partially explain the observed inability to complete oocyst development. However, the abundant *UIS12* mRNA levels in ookinetes suggest UIS12 presence at that stage and a potential additional checkpoint of post-transcriptional control by *UIS12* possibly after transcription of mosquito stage-specific target genes is orchestrated by a family of closely related Apetala-2 proteins, AP2-O 1-4, in ookinetes (Kaneko et al., 2015; Modrzynska et al., 2017). And this additional checkpoint in ookinetes might be the reason for the pleiotropic effects observed upon absence of *UIS12*, finally resulting in the aberrant maturation of oocysts. Due to the low *UIS12* transcript levels in oocysts a direct role at this point in the life cycle is less likely. We, thus, propose that UIS12 exerts multiple regulatory roles at consecutive life cycle stages, and, hence, is together with Puf2 one of the first known *Plasmodium* RNA binding proteins with pleiotropic functions for life cycle progression (Gomes-Santos et al., 2011; Lindner et al., 2013; Miao et al., 2013; Miao et al., 2010; Muller et al., 2011).

The GO-term enrichment of down-regulated transcripts in *uis12(-)* shows perturbation of many kinases and GO-term enrichment of up-regulated transcripts revealed perturbation of gene expression. A MEME search in the ORFs, 5’ UTRs and 3’ UTRs of the up-regulated transcripts identified in the microarray did reveal a 10-nucleotide U-rich motif in the ORFs unique to the analyzed subsets when compared to sequences that were not perturbated in absence of *UIS12*. This 10 nucleotide-long U-rich motif might represent a signature of UIS12 interaction with target mRNAs during blood infection, which might be required for proper preparation for host switching. We wish to point out that down-regulation of gametocyte signature transcripts in *uis12(-)* blood stages can be largely attributed to the at least 3.5-fold reduction in gametocyte numbers, yet the levels of down-regulation ranged from a low (-2, e.g., *Puf2*) to more than 15-fold (e.g., *CCP1* and *CCP2*). By qPCR we could confirm the down-regulation of some of the gametocyte specific transcripts such as P28 in a gametocyte enriched mRNA preparation of *uis12(-)* blood stage parasites, indicating that the down-regulation of gametocyte transcripts might not only be a consequence of the absence of gametocytes in *uis12(-)* mixed blood stages, but a direct result of a specific UIS12-dependent regulation.

**Figure 7:**
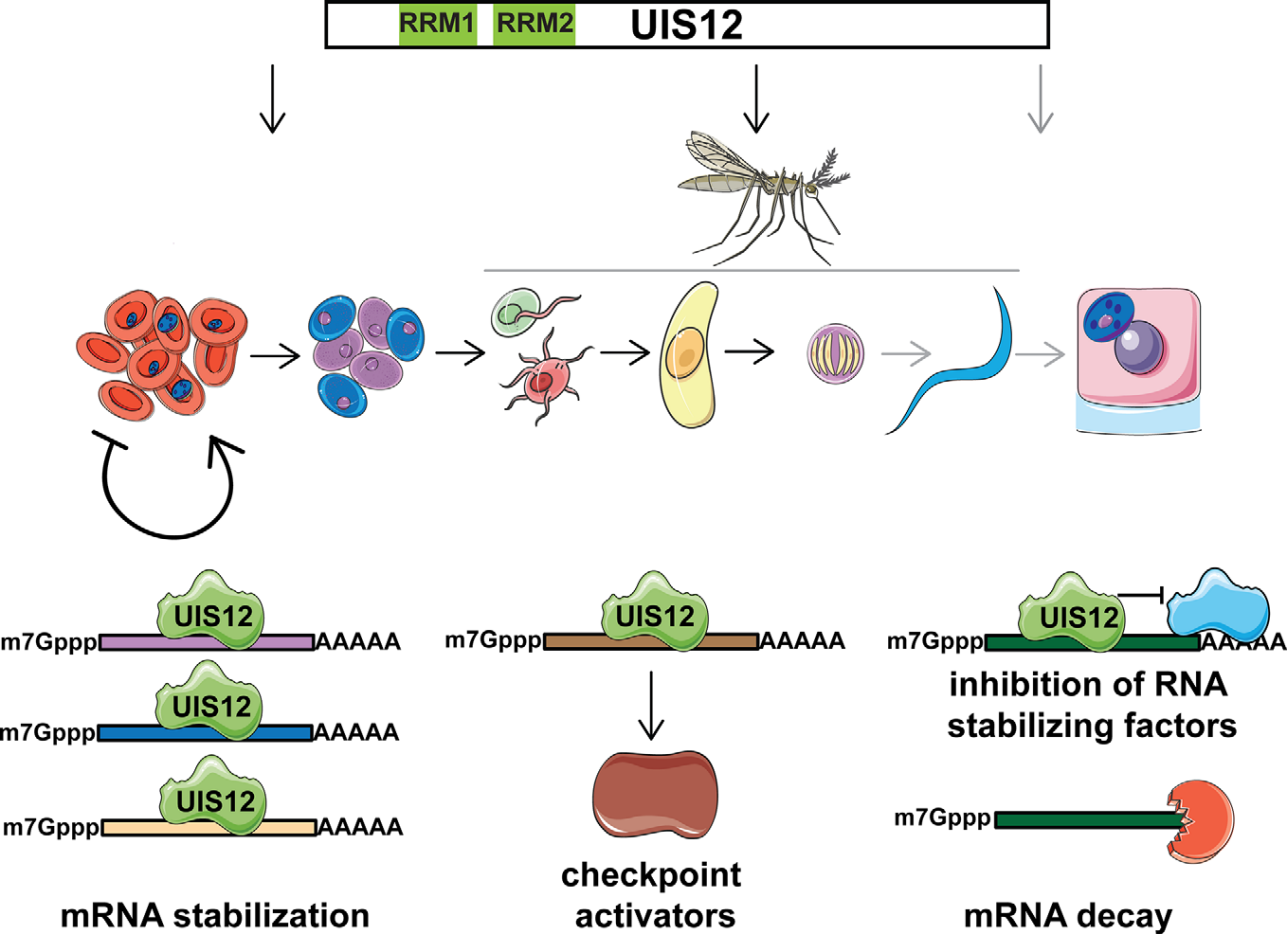
Potential roles of *Plasmodium* UIS12 in expression control of gametocyte- and mosquito stage-specific mRNAs. UIS12 appears to influence parasite stage conversion at three different points in the complex *Plasmodium* life cycle (top). During blood infection commitment to onward transmission is initiated and *Plasmodium* parasites undergo a series of developmental transitions (center); female (blue) and male gametocytes (purple) are formed in the mammalian host; inside the mosquito midgut male gametes (red) fertilize female gametes (green), leading to formation of a motile zygote, the ookinete (yellow); oocyst development leads to formation of sporozoites (blue), which upon another mosquito bite invade hepatocytes (pink) and form liver stages (blue). Note that the last checkpoint (grey) could not be addressed experimentally because of complete life cycle arrest prior to sporozoite formation. The candidate RNA-binding protein UIS12 might modulate expression of gametocyte- and mosquito stage-specific transcripts by one of at least three alternative mechanisms (bottom); i) stabilization and/or translational repression of a large set of target mRNAs (left); ii) binding and stabilization of few mRNAs that in turn encode checkpoint activators (center); iii) inhibition of partner protein(s), which in turn stabilize target mRNAs (right). In the absence of *UIS12* post-transcriptional expression of a large set of gametocyte and mosquito stage -specific genes is impaired resulting in reduced differentiation and, ultimately, arrest of life cycle progression.

Further analyses employing RNA-Seq or single cell transcriptomics on synchronized and purified stages, ranging from ring stages to ookinetes, are warranted to gain an in-depth understanding of the mRNA repertoire that is under UIS12 control. Since the shared signature motif was identified in up-regulated transcripts, this would argue against a role of UIS12 as an mRNA stabilizing factor. At least three alternative scenarios emerge (**Figure 7**). One potential, albeit unusual, mechanism of UIS12 could be binding and stabilizing of large set of target mRNAs that encode gametocyte- and mosquito stage-specific proteins. Alternatively, UIS12 acts on one or few mRNAs, which in turn drive expression control, for instance by stabilizing transcripts that encode checkpoint activators. A reverse, and perhaps more likely function would be an inhibitory role towards target mRNAs or a partner protein, which in turn modulate the half-lives of target mRNAs. According to such a scenario, up-regulation of transcripts in the absence of UIS12 might be a consequence of increased mRNA stability. We consider the identification of a nucleotide motif a good starting point for future work, and are hesitant to propose a molecular mechanism at this early stage of discovery.

In gametocytes, the conserved DOZI/CITH RNA storage complex (Mair et al., 2006; Mair et al., 2010; Guerreiro et al., 2014) is central to translational repression. For instance, the DOZI/CITH complex is involved in translationally repressing *P25*, *P28, PlasmepsinVI*, *AP2*-O, and *GAP45*. These transcripts are also contained on our list of down-regulated transcripts in *uis12(-)* blood stages. Thus, we were curious how many targets overlap when we compared the reported 117 genes down-regulated in both *dozi(-)* and *cith(-)* as determined by microarray on gametocytes (Mair et al., 2010) with the 646 down-regulated transcripts in *uis12(-)* blood stages (<-2-fold change), we found a surprisingly high overlap, of 102 of the 117 transcripts (**Figure 6B**). The RNA-helicase DOZI was previously shown to be a key regulator of *P25* and *P28* transcript stability, as well as of 731 other transcripts, and they all share a conserved U-rich nucleotide motif mainly in the 5’ and 3’ UTR (Hall et al., 2005; Braks et al., 2008; Guerreiro et al., 2014). The abundance of U residues in the DOZI and UIS12 motifs could hint at a related mechanism. The key differences are that the U-rich motif in our study is found in the ORFs of target genes, while the U-rich motif bound by the DOZI complex is located in 5’ and 3’ regions of its targets (Braks et al., 2008). Also, the U-rich motif identified for DOZI binding has a length of 47 nucleotides, whereas the motif identified herein is only 10 nucleotides long. Despite these clear distinctions a potential functional link of these two proteins remains an attractive possibility, since UIS12 and DOZI have >100 overlapping down-regulated targets (**Figure 6B**).

We note that, in marked contrast to *UIS12*, gene deletion of *DOZI* leads to a developmental arrest of the fertilized female gamete, a process that is not abolished in the absence of UIS12. In all three knockout lines, *uis12(-)*, *dozi(-)*, and *puf2(-)* parasites the mRNA levels of *P25* and *P28* were perturbated. While in *dozi(-)* and *uis12(-)* the transcripts were down-regulated, in *puf2(-)* an up-regulation was described, indicating involvement in distinct shared and opposing regulatory processes. On the other hand, PlasmepsinVI is translationally repressed by *Pf*Puf2 (Miao et al., 2013) and reduced in *uis12(-)* parasites, similar to the meiotic recombinase *DMC1* (Mlambo et al., 2012). Both knockouts as well as the *CCP* knockouts resemble the defects of *uis12(-)*. Clearly, further work is warranted to decipher the molecular mechanisms that orchestrate the sequential post-transcriptional control events and contributions of the regulatory molecules.

In conclusion, our experimental genetics analysis of the *Plasmodium berghei* candidate RNA binding protein *UIS12* uncovered broad perturbations of mRNA expression during blood infection, including up-regulation of several hundred transcripts, which share a U-rich 10 nucleotide-long motif in their open reading frames. The observed mRNA deregulation correlates with pleiotropic defects after blood stage propagation during sexual stage development, mosquito midgut colonization and oocyst maturation, ultimately leading to a complete block in the *Plasmodium* life cycle inside the *Anopheles* vector.

## Methods

### Ethics Statement

All animal work conducted in this study was carried out in strict accordance with the German “Tierschutzgesetz in der Fassung vom 18. Mai 2006 (BGBl. I S. 1207)”, which implements the directive 86/609/EEC from the European Union and the European Convention for the protection of vertebrate animals used for experimental and other scientific purposes. The protocol was approved by the ethics committee of MPI-IB and the Berlin state authorities (LAGeSo Reg# G0469/09, G0294/15).

### Parasites and Experimental Animals

*P. berghei* ANKA cl507 parasites that constitutively express GFP under the *Pb*EF1α promoter and *P. berghei* ANKA Bergreen parasites that express the green fluorescent protein GFP under the *Pb*HSP70 promoter throughout their life cycle have been used in our experiments (Franke-Fayard et al., 2004; Kooij et al., 2012).

All mouse experiments were performed with female NMRI or C57BL/6 mice purchased from Charles River Laboratories (Sulzfeld, Germany) or bred in house. NMRI mice were used for transfection experiments, blood stage infections, and transmission to *Anopheles stephensi* mosquitos. C57BL/6 mice were used for sporozoite and blood stage infections, exflagellation essays and ookinete cultures.

### Gene Deletion of *P. berghei UIS12*

The *P. berghei UIS12* gene deletion was performed by double crossover homologous recombination of a replacement pB3D-knockout plasmid (van Dijk et al., 1995). The gene knockout construct targeting the endogenous *UIS12* locus comprised a 647 bp *UIS12* 5’ UTR and a 524 bp *UIS12* 3’ UTR sequence flanking a *T. gondii DHFR*/TS pyrimethamine-resistance cassette. **Table S4** lists the oligonucleotides used for generation of the knockout construct. Prior to transfection, plasmids were linearized by restriction digest. A digest with *Sac*II and *Kpn*I produced a fragment consisting of the pyrimethamine resistance cassette flanked by the *UIS12* 5’ and 3’ UTRs. Transfection by electroporation of cultured schizonts was performed as previously described (Janse et al., 2006b). Two independent parasite lines, one in *P. berghei* Bergreen and one in *P. berghei* cl507 recipient lines were generated and phenotypically characterized. For genotyping of recombinant parasites, knockout- and wild-type-specific PCR fragments were amplified from genomic DNA of parental and clonal lines with the oligonucleotides listed in **Table S4**.

### Quantitative Real-Time PCR (qRT-PCR)

Quantitative real-time PCR was used to quantify *UIS12* gene expression and for comparing gene expression in *uis12(-)* and wild-type (WT) mixed blood stages and gametocytes. For mixed blood stage analyses, C57BL/6 mice were infected i.v. with 10,000 iRBC, and 7 days after infection whole blood was isolated. The gametocyte samples were generated by infecting phenylhydrazine-treated NMRI mice with 10^6^ iRBC. Three to four days later, when gametocytemia reached the highest levels, 12.5 mg sulfadiazine was supplemented to 1 liter of drinking water. Within 48 h the sulfadiazine treatment eliminated the asexual stages, which was confirmed by a Giemsa-stained thin blood film. Next, the infected blood was harvested. To isolate mixed blood stages or gametocytes, whole blood was cleaned from white blood cells and erythrocytes using cellulose-glass bead columns and saponin (0.3%; Sigma-Aldrich) lysis. Parasites were stored in 1 ml TRIzol (Invitrogen) or RLT-Buffer with β-mercaptoethanol (Qiagen). Parasite RNA was isolated with the RNeasy Kit (Qiagen) or TRIzol-Reagent (Sigma-Aldrich). DNase treatment with Quiagen RNase free DNase Set or Ambion Turbo DNA free Kit was performed to remove residual genomic DNA.

Reverse transcription to cDNA was done using the RETROscript Kit (Ambion). The qPCR was performed on parasite cDNA that was generated from total RNA of selected *P. berghei* life cycle stages. Quantitative real-time PCR was performed as previously described (Silvie et al., 2008) using Power SYBR Green PCR Master Mix and the StepOnePlus Real-Time PCR System (both Applied Biosystems). All procedures were performed according to the manufacturer’s instructions. Life cycle expression data were normalized to GFP that is constitutively expressed under the *EF1α* promoter in *P. berghei* 507cl. Mixed blood stage and gametocyte expression data for comparison of knockout and WT parasites were normalized to *PbHSP70*. The relative transcript abundance was determined by the 2^ΔΔCT^ method. For oligonucleotide sequences refer to **Table S4**.

### *Plasmodium* Life Cycle: Parasite Asexual and Sexual Growth, Mosquito Infection, and Sporozoite Transmission

For asexual blood stage and gametocyte growth curves, C57BL/6 mice were injected intravenously with 10,000 infected red blood cells. From day 3 to 7, Giemsa (VWR)-stained thin blood films were microscopically examined to determine parasitemia and gametocytemia. Parasitemia and gametocytemia are defined as the percentage of red blood cells infected with asexual stages or gametocytes, respectively.

To analyze male exflagellation, C57BL/6 mice were intravenously injected with 10^7^ infected red blood cells (iRBC). After four days, parasitemia and gametocytemia were determined. Next, five microliters of tail blood were diluted 1:25 in ookinete medium (RPMI 1640, 10% FCS and 50 mM xanthurenic acid, pH 8.0) and incubated for 12 min at room temperature. For the next 6 min exflagellation centers were counted with help of a Neubauer chamber.

Ookinete cultures were set up as previously described (Weiss et al., 1997). Four days after the intravenous injection of 10^6^ iRBC into C57BL/6 mice pre-treated one day earlier with phenylhydrazine 97% (Sigma-Aldrich, 100µl of 6.18 µl/ml) 1 ml of fresh mouse blood was mixed with 10 ml of ookinete medium. After 20 h incubation at 20°C, at 80% humidity the culture was examined for development of ookinetes and ookinetes were purified with anti-P28-coupled magnetic beads.

*Anopheles stephensi* mosquitoes were kept at 28°C (non-infected) or 20°C (infected) under a 14 h light/10 h dark cycle at 80% humidity. Four days after the transfer of 10^7^ iRBC, when exflagellation reached a maximum, mosquitoes were infected as described (Vanderberg, 1975). After 4 to 14 days, midguts were isolated and oocyst development analyzed by fluorescent microscopy (Zeiss AxioObserver Z1). For visualization of nuclei, HOECHST 33342 (Invitrogen) was added at a 1:1,000 dilution. Images were analyzed using the FIJI processing (oocyst diameter) and/or Photoshop (oocyst numbers) software.

Midguts and salivary glands were analyzed at day 14–18 and day 18–30, respectively, for presence of sporozoites.

To test *Plasmodium* transmission, age-matched female C57BL/6 mice were either subjected to the bites of ~100 infected mosquitos or inoculated with 10,000 sporozoites, or for *uis12(-)* the salivary gland extract of 20 mosquitos, in RPMI intravenously. After three days Giemsa-stained thin blood films were analyzed for presence of infected red blood cells.

### Microarray Expression Profiling of *P. berghei* Blood Stages

The microarray was performed on two biological replicate samples of *uis12(-)* and wild-type with comparable parasitemia. One replicate was generated by infecting one C57BL/6 mouse each with 10,000 *uis12(-)* or wild-type blood stages intravenously (R1, biological replicate 1). After 7 days of infection, mixed blood stages were isolated from whole blood. For both samples, the parasitemia was comparable (*uis12(-)* 6.3%, WT 6.2%). The other replicate (R2, biological replicate 2) was generated by injecting one NMRI mouse with 10^7^ *uis12(-)* or wild-type blood stages and mixed blood stage parasites were harvested after 3 days (*uis12(-)* 8.7%, WT 6.4%).

Whole blood was isolated and cleaned from white blood cells and erythrocytes using cellulose-glass bead columns and saponin (0.3%; Sigma-Aldrich) lysis. About 10^8^ parasites were stored in 1ml TRIzol (Invitrogen). Total RNA was isolated using glycogen as a carrier according to the ‘TRIzol Reagent RNA preparation method’ (Invitrogen). The amount of RNA was determined by OD 260/280 nm measurement using a ‘NanoDrop 1000’ (peQlab) spectrophotometer. The RNA size, integrity and the amount of total RNA were measured with a ‘Bioanalyzer 2100’ using an ‘RNA Nano 6000 Microfluidics Kit’ (both Agilent genomics). RNA labeling was performed with the two color ‘Quick Amp Labeling Kit’ (Agilent genomics) using a 1:5 mixture of ‘FullSpectrum MultiStart Primer’ (Systembio). mRNA was reverse transcribed and amplified using the primer mixture. The RNA was split and labeled with Cyanine 3-CTP and Cyanine 5-CTP, respectively. After precipitation, purification and quantification, labeled samples were hybridized to the Agilent ‘4x44K custom-commercial microarrays’ according to the manufacturers protocol. Microarray experiments were performed as dual-color hybridizations. A color-swap dye-reversal was performed in order to compensate specific effects of the dyes and to ensure statistically relevant data analysis (Churchill, 2002; Wernersson et al., 2007). Scanning of the microarrays was performed with 5 µm resolution and the extended mode using a ‘High Resolution Microarray Laser Scanner’ (G2505, Agilent Technologies). Raw microarray image data were extracted and analyzed with the ‘G2567AA Image Analysis/Feature Extraction software’ (Version A.10.5.1.1, Agilent Technologies). On reporter and sequence level, the extracted MAGE-ML files were further analyzed with the ‘Rosetta Resolver Biosoftware’ (7.2.2.0 SP1.31). All subsequent data analysis was performed with the Microsoft Excel, Prism and the PlasmoDB (http://plasmodb.org/plasmo/) database. *P. berghei* microarrays were designed on the OligoWiz (http://www.cbs.dtu.dk/services/OligoWiz/) server that included annotated open reading frames (ORF) from the whole genome of *P. berghei*. ORF specific probes were designed as sense oligonucleotides of the coding strand (Wernersson et al., 2007). The aimed oligonucleotide length was between 50 and 60 bases. To avoid self-hit, the maximal homology was 97%, the cross hybridization maximum length was at 80% and random primed position preference scores within OligoWiz were chosen. Depending on these parameters and the sequence input length, each ORF was covered by different numbers of specific oligonucleotide probes. In total 41,133 specific probe sequences were uploaded to eArray (Agilent Technologies, https://earray.chem.agilent.com/earray/) as expression application with a customer specified feature layout.

The microarray data discussed in this publication have been deposited in NCBI’s Gene Expression Omnibus (Edgar et al., 2002) and are accessible through GEO Series accession number GSE152686.

### Statistical Analysis and Online Bioinformatic Tools

Statistical significance was determined with the help of Graph Pad Prism software, using Mann-Whitney test for comparison of parasitemia, gametocytemia, and exflagellation dot-plots and multiple t-tests (one per row) for all growth curves.

The identification and positioning of the two RRM domains of the UIS12 orthologous proteins has been performed by NCBI WEB batch-CD Search Tool (Marchler-Bauer et al., 2015). Protein comparison was done by NCBI protein blast (blastp) with default settings, no compositional adjustment and without low complexity filter. Sequence coverage of all *Plasmodium* orthologues was higher than 90%.

Gene ontology (GO) enrichment analysis (Ashburner et al., 2000) was performed with help of the Gene Ontology enrichment analysis tool from PlasmoDB, with the default settings and the redundant GO terms were removed with help of the REVIGO software.

To screen for common 8-10 nucleotide-long motifs we used the MEME webtool (Bailey et al., 2009), screened all ORFs, 5’UTR sequences and 3’UTR sequences (1,000 nucleotides per gene for ORFs) of transcripts up-regulated >2.5-fold and compared them to the respective gene parts of the non-regulated (-1.5 to -1.5) transcripts from our microarray analysis of biological replicate 1.

Parts of the illustrations used in **Figure 7** were obtained from Servier Medical Art, (http://www.servier.fr/servier-medical-art) licensed under a Creative Commons Attribution 3.0 Unported License.

## Data Availability Statement

The microarray data discussed in this publication have been deposited in NCBI’s Gene Expression Omnibus (Edgar et al., 2002) and are accessible through GEO Series accession number GSE152686 (https://www.ncbi.nlm.nih.gov/geo/query/acc.cgi?acc=GSE152686).

## Ethics Statement

The animal study was reviewed and approved by the ethics committee of MPI-IB and the Berlin state authorities (LAGeSo Reg# G0469/09, G0294/15).

## Author Contributions

KMa, OS, and KMü designed the experiments. KMü performed experiments and analyzed data. H-JM performed and analyzed the microarray. KMa and KMü wrote the paper. All authors commented on and revised the manuscript. All authors contributed to the article and approved the submitted version.

## Funding

This work was funded in part by the Max Planck Society and the DFG.

## Conflict of Interest

The authors declare that the research was conducted in the absence of any commercial or financial relationships that could be construed as a potential conflict of interest.

## References

[B1] Ashburner M. Ball C. A. Blake J. A. Botstein D. Butler H. Cherry J. M.. (2000). Gene ontology: tool for the unification of biology. The Gene Ontology Consortium. Nat. Genet. 25, 25–29. 10.1038/75556 10802651 PMC3037419

[B2] Bailey T. L. Boden M. Buske F. A. Frith M. Grant C. E. Clementi L.. (2009). MEME SUITE: tools for motif discovery and searching. Nucleic Acids Res. 37, W202–W208. 10.1093/nar/gkp335 19458158 PMC2703892

[B3] Balaji S. Babu M. M. Iyer L. M. Aravind L. (2005). Discovery of the principal specific transcription factors of Apicomplexa and their implication for the evolution of the AP2-integrase DNA binding domains. Nucleic Acids Res. 33, 3994–4006. 10.1093/nar/gki709 16040597 PMC1178005

[B4] Billker O. Lindo V. Panico M. Etienne A. E. Paxton T. Dell A.. (1998). Identification of xanthurenic acid as the putative inducer of malaria development in the mosquito. Nature 392, 289–292. 10.1038/32667 9521324

[B5] Billker O. Dechamps S. Tewari R. Wenig G. Franke-Fayard B. Brinkmann V. (2004). Calcium and a calcium-dependent protein kinase regulate gamete formation and mosquito transmission in a malaria parasite. Cell 117, 503–514. 10.1016/S0092-8674(04)00449-0 15137943

[B6] Braks J. A. Mair G. R. Franke-Fayard B. Janse C. J. Waters A. P. (2008). A conserved U-rich RNA region implicated in regulation of translation in *Plasmodium* female gametocytes. Nucleic Acids Res. 36, 1176–1186. 10.1093/nar/gkm1142 18158300 PMC2275103

[B7] Carter V. Nacer A. M. Underhill A. Sinden R. E. Hurd H. (2007). Minimum requirements for ookinete to oocyst transformation in *Plasmodium* . Int. J. Parasitol. 37, 1221–1232. 10.1016/j.ijpara.2007.03.005 17482621 PMC2474741

[B8] Churchill G. A. (2002). Fundamentals of experimental design for cDNA microarrays. Nat. Genet. 32 (Suppl), 490–495. 10.1038/ng1031 12454643

[B9] Deligianni E. Morgan R. N. Bertuccini L. Kooij T. W. Laforge A. Nahar C.. (2011). Critical role for a stage-specific actin in male exflagellation of the malaria parasite. Cell Microbiol. 13, 1714–1730. 10.1111/j.1462-5822.2011.01652.x 21790945

[B10] Dreyfuss G. Swanson M. S. Pinol-Roma S. (1988). Heterogeneous nuclear ribonucleoprotein particles and the pathway of mRNA formation. Trends Biochem. Sci. 13, 86–91. 10.1016/0968-0004(88)90046-1 3072706

[B11] Edgar R. Domrachev M. Lash A. E. (2002). Gene Expression Omnibus: NCBI gene expression and hybridization array data repository. Nucleic Acids Res. 30, 207–210. 10.1093/nar/30.1.207 11752295 PMC99122

[B12] Franke-Fayard B. Trueman H. Ramesar J. Mendoza J. van der Keur M. van der Linden R.. (2004). A *Plasmodium berghei* reference line that constitutively expresses GFP at a high level throughout the complete life cycle. Mol. Biochem. Parasitol. 137, 23–33. 10.1016/j.molbiopara.2004.04.007 15279948

[B13] Gomes-Santos C. S. Braks J. Prudencio M. Carret C. Gomes A. R. Pain A.. (2011). Transition of *Plasmodium* sporozoites into liver stage-like forms is regulated by the RNA binding protein Pumilio. PloS Pathog. 7, e1002046. 10.1371/journal.ppat.1002046 21625527 PMC3098293

[B14] Guerreiro A. Deligianni E. Santos J. M. Silva P. A. Louis C. Pain A.. (2014). Genome-wide RIP-Chip analysis of translational repressor-bound mRNAs in the *Plasmodium* gametocyte. Genome Biol. 15, 493. 10.1186/s13059-014-0493-0 25418785 PMC4234863

[B15] Hall N. Karras M. Raine J. D. Carlton J. M. Kooij T. W. Berriman M.. (2005). A comprehensive survey of the *Plasmodium* life cycle by genomic, transcriptomic, and proteomic analyses. Science 307, 82–86. 10.1126/science.1103717 15637271

[B16] Howick V. M. Russell A. J. C. Andrews T. Heaton H. Reid A. J. Natarajan K.. (2019). The Malaria Cell Atlas: Single parasite transcriptomes across the complete *Plasmodium* life cycle. Science 365, eaaw2619. 10.1126/science.aaw2619 31439762 PMC7056351

[B17] Invergo B. M. Brochet M. Yu L. Choudhary J. Beltrao P. Billker O. (2017). Sub-minute phosphoregulation of cell cycle systems during *Plasmodium* gamete formation. Cell Rep. 21, 2017–2029. 10.1016/j.celrep.2017.10.071 29141230 PMC5700370

[B18] Janse C. J. Franke-Fayard B. Waters A. P. (2006a). Selection by flow-sorting of genetically transformed, GFP-expressing blood stages of the rodent malaria parasite, *Plasmodium berghei* . Nat. Protoc. 1, 614–623. 10.1038/nprot.2006.88 17406288

[B19] Janse C. J. Ramesar J. Waters A. P. (2006b). High-efficiency transfection and drug selection of genetically transformed blood stages of the rodent malaria parasite *Plasmodium berghei* . Nat. Protoc. 1, 346–356. 10.1038/nprot.2006.53 17406255

[B20] Josling G. A. Russell T. J. Venezia J. Orchard L. van Biljon R. Painter H. J.. (2020). Dissecting the role of PfAP2-G in malaria gametocytogenesis. Nat. Commun. 11, 1503. 10.1038/s41467-020-15026-0 32198457 PMC7083873

[B21] Kaneko I. Iwanaga S. Kato T. Kobayashi I. Yuda M. (2015). Genome-wide identification of the target genes of AP2-O, a *Plasmodium* AP2-family transcription factor. PloS Pathog. 11, e1004905. 10.1371/journal.ppat.1004905 26018192 PMC4446032

[B22] Kooij T. W. Rauch M. M. Matuschewski K. (2012). Expansion of experimental genetics approaches for *Plasmodium berghei* with versatile transfection vectors. Mol. Biochem. Parasitol. 185, 19–26. 10.1016/j.molbiopara.2012.06.001 22705315

[B23] Lal K. Delves M. J. Bromley E. Wastling J. M. Tomley F. M. Sinden R. E. (2009). *Plasmodium* male development gene-1 (mdv-1) is important for female sexual development and identifies a polarised plasma membrane during zygote development. Int. J. Parasitol. 39, 755–761. 10.1016/j.ijpara.2008.11.008 19136003

[B24] Lasonder E. Janse C. J. van Gemert G. J. Mair G. R. Vermunt A. M. Douradinha B. G.. (2008). Proteomic profiling of *Plasmodium* sporozoite maturation identifies new proteins essential for parasite development and infectivity. PloS Pathog. 4, e1000195. 10.1371/journal.ppat.1000195 18974882 PMC2570797

[B25] Lasonder E. Rijpma S. R. van Schaijk B. C. Hoeijmakers W. A. Kensche P. R. Gresnigt M. S.. (2016). Integrated transcriptomic and proteomic analyses of *P. falciparum* gametocytes: molecular insight into sex-specific processes and translational repression. Nucleic Acids Res. 44, 6087–6101. 10.1093/nar/gkw536 27298255 PMC5291273

[B26] Lavazec C. Moreira C. K. Mair G. R. Waters A. P. Janse C. J. Templeton T. J. (2009). Analysis of mutant *Plasmodium berghei* parasites lacking expression of multiple PbCCp genes. Mol. Biochem. Parasitol. 163, 1–7. 10.1016/j.molbiopara.2008.09.002 18848846

[B27] Le Roch K. G. Johnson J. R. Florens L. Zhou Y. Santrosyan A. Grainger M.. (2004). Global analysis of transcript and protein levels across the *Plasmodium falciparum* life cycle. Genome Res. 14, 2308–2318. 10.1101/gr.2523904 15520293 PMC525690

[B28] Lindner S. E. Mikolajczak S. A. Vaughan A. M. Moon W. Joyce B. R. Sullivan W. J. Jr.. (2013). Perturbations of *Plasmodium* Puf2 expression and RNA-seq of Puf2-deficient sporozoites reveal a critical role in maintaining RNA homeostasis and parasite transmissibility. Cell Microbiol. 15, 1266–1283. 10.1111/cmi.12116 23356439 PMC3815636

[B29] Lindner S. E. Swearingen K. E. Shears M. J. Walker M. P. Vrana E. N. Hart K. J.. (2019). Transcriptomics and proteomics reveal two waves of translational repression during the maturation of malaria parasite sporozoites. Nat. Commun. 10, 4964. 10.1038/s41467-019-12936-6 31673027 PMC6823429

[B30] Mair G. R. Braks J. A. Garver L. S. Wiegant J. C. Hall N. Dirks R. W.. (2006). Regulation of sexual development of *Plasmodium* by translational repression. Science 313, 667–669. 10.1126/science.1125129 16888139 PMC1609190

[B31] Mair G. R. Lasonder E. Garver L. S. Franke-Fayard B. M. Carret C. K. Wiegant J. C.. (2010). Universal features of post-transcriptional gene regulation are critical for *Plasmodium* zygote development. PloS Pathog. 6, e1000767. 10.1371/journal.ppat.1000767 20169188 PMC2820534

[B32] Marchler-Bauer A. Derbyshire M. K. Gonzales N. R. Lu S. Chitsaz F. Geer L. Y.. (2015). CDD: NCBI’s conserved domain database. Nucleic Acids Res. 43, D222–D226. 10.1093/nar/gku1221 25414356 PMC4383992

[B33] Maris C. Dominguez C. Allain F. H. (2005). The RNA recognition motif, a plastic RNA-binding platform to regulate post-transcriptional gene expression. FEBS J. 272, 2118–2131. 10.1111/j.1742-4658.2005.04653.x 15853797

[B34] Matuschewski K. Ross J. Brown S. M. Kaiser K. Nussenzweig V. Kappe S. H. (2002). Infectivity-associated changes in the transcriptional repertoire of the malaria parasite sporozoite stage. J. Biol. Chem. 277, 41948–41953. 10.1074/jbc.M207315200 12177071

[B35] Matuschewski K. (2006). Getting infectious: formation and maturation of *Plasmodium* sporozoites in the Anopheles vector. Cell Microbiol. 8, 1547–1556. 10.1111/j.1462-5822.2006.00778.x 16984410

[B36] Miao J. Li J. Fan Q. Li X. Li X. Cui L. (2010). The Puf-family RNA-binding protein PfPuf2 regulates sexual development and sex differentiation in the malaria parasite *Plasmodium falciparum* . J. Cell Sci. 123, 1039–1049. 10.1242/jcs.059824 20197405 PMC2844316

[B37] Miao J. Fan Q. Parker D. Li X. Li J. Cui L. (2013). Puf mediates translation repression of transmission-blocking vaccine candidates in malaria parasites. PloS Pathog. 9, e1003268. 10.1371/journal.ppat.1003268 23637595 PMC3630172

[B38] Mlambo G. Coppens I. Kumar N. (2012). Aberrant sporogonic development of Dmc1 (a meiotic recombinase) deficient *Plasmodium berghei* parasites. PloS One 7, e52480. 10.1371/journal.pone.0052480 23285059 PMC3528682

[B39] Modrzynska K. Pfander C. Chappell L. Yu L. Suarez C. Dundas K.. (2017). A knockout screen of ApiAP2 genes reveals networks of interacting transcriptional regulators controlling the *Plasmodium* life cycle. Cell Host Microbe 21, 11–22. 10.1016/j.chom.2016.12.003 28081440 PMC5241200

[B40] Muller K. Matuschewski K. Silvie O. (2011). The Puf-family RNA-binding protein Puf2 controls sporozoite conversion to liver stages in the malaria parasite. PloS One 6, e19860. 10.1371/journal.pone.0019860 21673790 PMC3097211

[B41] Otto T. D. Bohme U. Jackson A. P. Hunt M. Franke-Fayard B. Hoeijmakers W. A.. (2014). A comprehensive evaluation of rodent malaria parasite genomes and gene expression. BMC Biol. 12, 86. 10.1186/s12915-014-0086-0 25359557 PMC4242472

[B42] Pace T. Olivieri A. Sanchez M. Albanesi V. Picci L. Siden Kiamos I.. (2006). Set regulation in asexual and sexual *Plasmodium* parasites reveals a novel mechanism of stage-specific expression. Mol. Microbiol. 60, 870–882. 10.1111/j.1365-2958.2006.05141.x 16677299

[B43] Painter H. J. Campbell T. L. Llinas M. (2011). The Apicomplexan AP2 family: integral factors regulating *Plasmodium* development. Mol. Biochem. Parasitol. 176, 1–7. 10.1016/j.molbiopara.2010.11.014 21126543 PMC3026892

[B44] Pradel G. Hayton K. Aravind L. Iyer L. M. Abrahamsen M. S. Bonawitz A.. (2004). A multidomain adhesion protein family expressed in *Plasmodium falciparum* is essential for transmission to the mosquito. J. Exp. Med. 199, 1533–1544. 10.1084/jem.20031274 15184503 PMC2211786

[B45] Raine J. D. Ecker A. Mendoza J. Tewari R. Stanway R. R. Sinden R. E. (2007). Female inheritance of malarial lap genes is essential for mosquito transmission. PloS Pathog. 3, e30. 10.1371/journal.ppat.0030030 17335349 PMC1808070

[B46] Reddy B. P. Shrestha S. Hart K. J. Liang X. Kemirembe K. Cui L.. (2015). A bioinformatic survey of RNA-binding proteins in *Plasmodium* . BMC Genomics 16, 890. 10.1186/s12864-015-2092-1 26525978 PMC4630921

[B47] Saeed S. Carter V. Tremp A. Z. Dessens J. T. (2010). *Plasmodium berghei* crystalloids contain multiple LCCL proteins. Mol. Biochem. Parasitol. 170, 49–53. 10.1016/j.molbiopara.2009.11.008 19932717 PMC2816727

[B48] Saeed S. Carter V. Tremp A. Z. Dessens J. T. (2013). Translational repression controls temporal expression of the *Plasmodium berghei* LCCL protein complex. Mol. Biochem. Parasitol. 189, 38–42. 10.1016/j.molbiopara.2013.04.006 23684590 PMC3694310

[B49] Shrestha S. Li X. Ning G. Miao J. Cui L. (2016). The RNA-binding protein Puf1 functions in the maintenance of gametocytes in *Plasmodium falciparum* . J. Cell Sci. 129, 3144–3152. 10.1242/jcs.186908 27383769 PMC5004898

[B50] Siden-Kiamos I. Vlachou D. Margos G. Beetsma A. Waters A. P. Sinden R. E.. (2000). Distinct roles for pbs21 and pbs25 in the *in vitro* ookinete to oocyst transformation of *Plasmodium berghei* . J. Cell Sci. 113, 3419–3426. 10984433 10.1242/jcs.113.19.3419

[B51] Silvie O. Goetz K. Matuschewski K. (2008). A sporozoite asparagine-rich protein controls initiation of *Plasmodium* liver stage development. PloS Pathog. 4, e1000086. 10.1371/journal.ppat.1000086 18551171 PMC2398788

[B52] Sinha A. Hughes K. R. Modrzynska K. K. Otto T. D. Pfander C. Dickens N. J.. (2014). A cascade of DNA-binding proteins for sexual commitment and development in *Plasmodium* . Nature 507, 253–257. 10.1038/nature12970 24572359 PMC4105895

[B53] Tomas A. M. Margos G. Dimopoulos G. van Lin L. H. de Koning-Ward T. F. Sinha R. (2001). P25 and P28 proteins of the malaria ookinete surface have multiple and partially redundant functions. EMBO J. 20, 3975–3983. 10.1093/emboj/20.15.3975 11483501 PMC149139

[B54] Trueman H. E. Raine J. D. Florens L. Dessens J. T. Mendoza J. Johnson J.. (2004). Functional characterization of an LCCL-lectin domain containing protein family in *Plasmodium berghei* . J. Parasitol. 90, 1062–1071. 10.1645/GE-3368 15562607

[B55] van Dijk M. R. Waters A. P. Janse C. J. (1995). Stable transfection of malaria parasite blood stages. Science 268, 1358–1362. 10.1126/science.7761856 7761856

[B56] van Dijk M. R. Janse C. J. Thompson J. Waters A. P. Braks J. A. Dodemont H. J.. (2001). A central role for P48/45 in malaria parasite male gamete fertility. Cell 104, 153–164. 10.1016/S0092-8674(01)00199-4 11163248

[B57] Vanderberg J. P. (1975). Development of infectivity by the *Plasmodium berghei* sporozoite. J. Parasitol. 61, 43–50. 10.2307/3279102 1090717

[B58] Vervenne R. A. Dirks R. W. Ramesar J. Waters A. P. Janse C. J. (1994). Differential expression in blood stages of the gene coding for the 21-kilodalton surface protein of ookinetes of *Plasmodium berghei* as detected by RNA in situ hybridisation. Mol. Biochem. Parasitol. 68, 259–266. 10.1016/0166-6851(94)90170-8 7739671

[B59] Weiss G. Thuma P. E. Mabeza G. Werner E. R. Herold M. Gordeuk V. R. (1997). Modulatory potential of iron chelation therapy on nitric oxide formation in cerebral malaria. J. Infect. Dis. 175, 226–230. 10.1093/infdis/175.1.226 8985227

[B60] Wernersson R. Juncker A. S. Nielsen H. B. (2007). Probe selection for DNA microarrays using OligoWiz. Nat. Protoc. 2, 2677–2691. 10.1038/nprot.2007.370 18007603

[B61] Yeoh L. M. Goodman C. D. Mollard V. McFadden G. I. Ralph S. A. (2017). Comparative transcriptomics of female and male gametocytes in *Plasmodium berghei* and the evolution of sex in alveolates. BMC Genomics 18, 734. 10.1186/s12864-017-4100-0 28923023 PMC5604118

[B62] Yuda M. Iwanaga S. Kaneko I. Kato T. (2015). Global transcriptional repression: An initial and essential step for *Plasmodium* sexual development. Proc. Natl. Acad. Sci. U.S.A. 112, 12824–12829. 10.1073/pnas.1504389112 26417110 PMC4611670

[B63] Yuda M. Kaneko I. Iwanaga S. Murata Y. Kato T. (2020). Female-specific gene regulation in malaria parasites by an AP2-family transcription factor. Mol. Microbiol. 113, 40–51. 10.1111/mmi.14334 31231888

[B64] Zhang M. Fennell C. Ranford-Cartwright L. Sakthivel R. Gueirard P. Meister S.. (2010). The *Plasmodium* eukaryotic initiation factor-2alpha kinase IK2 controls the latency of sporozoites in the mosquito salivary glands. J. Exp. Med. 207, 1465–1474. 10.1084/jem.20091975 20584882 PMC2901070

