# Supplementary material for: Pleiotropic Roles for the Plasmodium berghei RNA Binding Protein UIS12 in Transmission and Oocyst Maturation
Source: Front Cell Infect Microbiol. 2021 Mar 5;11:624945. doi: 10.3389/fcimb.2021.624945 (PMC7973279; doi:10.3389/fcimb.2021.624945)
Supplement: Supplementary file 1 [file DataSheet_1.pdf]

Supplementary Material

Pleiotropic Roles for the *Plasmodium berghei* RNA Binding Protein UIS12 in Transmission and Oocyst Maturation

Katja Müller, Olivier Silvie, Hans-Joachim Mollenkopf, and Kai Matuschewski

Contents:

- **Table S1 - S4**
- **Figures S1 and S2**

Table S1: The top 25 up-regulated genes in blood stages of *uis12(-) vs.* wild-type.

Gene ID	Product description	Mean -fold change R1[a] R2[b]	-fold change R1[a]	-fold change R2[b]
PBANKA_0216061	PIR protein	15.1	20.6	9.6
PBANKA_1000031	PIR protein	7.8	10.4	5.2
PBANKA_1428200	unknown	5.0	7.6	2.4
PBANKA_1100700	fam-a protein	4.9	5.2	4.6
PBANKA_0205900	candidate Niemann-Pick type C1-related protein	4.8	8.4	1.2
PBANKA_1100441	fam-b protein	3.7	5.0	2.5
PBANKA_1039600	RPN6 (26S proteasome regulatory subunit)	3.7	5.8	1.6
PBANKA_1034900	candidate pre-mRNA-splicing factor 38B	3.4	5.1	1.6
PBANKA_0524000	unknown	3.4	5.4	1.3
PBANKA_1221300	unknown	3.3	4.6	2.0
PBANKA_0211000	TIM50 (mitochondrial import inner membrane translocase subunit)	3.3	5.4	1.2
PBANKA_0722801	fam-b protein	3.2	4.5	1.9
PBANKA_1129300	YTM1 (ribosomal protein)	3.1	4.8	1.4
PBANKA_1013500	candidate large subunit GTPase 1	3.0	4.8	1.3
PBANKA_1146761	PIR protein	2.9	3.5	2.4
PBANKA_1012100	candidate HP12 protein homolog	2.9	4.4	1.4
PBANKA_1460800	candidate U3 small nucleolar RNA-associated protein 11	2.9	4.6	1.2
PBANKA_1218100	Cap380 (oocyst capsule protein)	2.9	4.2	1.6
PBANKA_1459800	unknown	2.9	4.2	1.5
PBANKA_0813200	candidate zinc finger protein	2.9	4.3	1.4
PBANKA_0900900	candidate reticulocyte binding protein	2.8	3.0	2.6
PBANKA_0704100	candidate ubiquitin conjugation factor E4 B	2.8	4.3	1.2
PBANKA_0715600	candidate 26S protease regulatory subunit 6B	2.8	3.9	1.6
PBANKA_1404500	unknown	2.8	2.8	2.7
PBANKA_0915500	glycine cleavage system H protein	2.7	3.9	1.5

[a] biological replicate 1

[b] biological replicate 2

Table S2: GO term enrichment analysis of biological processes of transcripts down-regulated in *uis12(-)*.

	GO term	Total number of genes with this GO term	Number of transcripts down-regulated[a] in *uis12(-)* with this GO term	Fold enrich-ment	Odds ratio[b]	P-value[c]
GO:0030031	cell projection assembly	2	2	9.0	>100	0.01
GO:0030030	cell projection organization	2	2	9.0	>100	0.01
GO:0044089	positive regulation of cellular component biogenesis	3	3	9.0	>100	0.01
GO:1902905	positive regulation of supramolecular fiber organization	3	3	9.0	>100	0.01
GO:0007131	reciprocal meiotic recombination	2	2	9.0	>100	0.01
GO:0000003	reproduction	2	2	9.0	>100	0.01
GO:0022414	reproductive process	2	2	9.0	>100	0.01
GO:0048869	cellular developmental process	4	3	9.0	>100	0.005
GO:0032502	developmental process	4	3	6.8	24.4	0.005
GO:0019673	GDP-mannose metabolic process	3	2	6.8	24.4	0.03
GO:0032956	regulation of actin cytoskeleton organization	6	4	6.0	16.2	0.002
GO:0090066	regulation of anatomical structure size	6	4	6.0	16.3	0.002
GO:0044087	regulation of cellular component biogenesis	6	4	6.0	16.3	0.002
GO:0032535	regulation of cellular component size	6	4	6.0	16.3	0.002
GO:0071976	cell gliding	17	11	6.0	16.3	1,7 e-8
GO:0046068	cGMP metabolic process	5	3	5.9	15.2	0.01
GO:0006928	movement of cell or subcellular component	50	30	5.4	12.2	3.4 e-17
GO:0051130	positive regulation of cellular component organization	5	3	5.4	13.2	0.01
GO:0009187	cyclic nucleotide metabolic process	9	5	5.4	12.2	0.001
GO:0051674	localization of cell	32	17	5.0	10.2	4 e-9
GO:0007017	microtubule-based process	38	20	4.8	9.6	2.2 e-10
GO:0040011	locomotion	47	24	4.8	9.5	7.4 e-12
GO:0009605	response to external stimulus	6	3	4.6	9.0	0.02
GO:0022402	cell cycle process	11	5	4.5	8.1	0.004
GO:0000280	nuclear division	7	3	4.1	6.8	0.03
GO:0035556	intracellular signal transduction	12	5	3.9	6.1	0.007

GO:0051128	regulation of cellular component organization	12	5	3.8	5.8	0.007
GO:0007010	cytoskeleton organization	18	7	3.8	5.8	0.002
GO:0051301	cell division	8	3	3.5	5.2	0.049
GO:0007049	cell cycle	19	7	3.4	4.9	0.003
GO:0048518	positive regulation of biological process	11	4	3.3	4.8	0.03
GO:0018345	protein palmitoylation	11	4	3.3	4.6	0.03
GO:0023052	signaling	22	8	3.3	4.6	0.002
GO:0007154	cell communication	23	8	3.3	4.7	0.002
GO:0044419	interspecies interaction between organisms	80	25	3.1	4.4	0.0000006
GO:0051704	multi-organism process	80	25	2.8	3.9	0.0000006
GO:0006468	protein phosphorylation	78	20	2.8	3.9	0.0002
GO:0016310	phosphorylation	99	23	2.3	2.9	0.0003
GO:0051716	cellular response to stimulus	71	13	2.1	2.6	0.04
GO:0006793	phosphorus metabolic process	200	32	1.7	1.8	0.02
GO:0050789	regulation of biological process	165	26	1.5	1.6	0.04
GO:0065007	biological regulation	185	28	1.4	1.6	0.048

[a] mean -fold change values of biological replicate 1 and 2 were used for this GO term enrichment analysis

[b] odds ratio statistics was calculated by Fisher's exact test

[c] *P*-values are from Fisher's exact test

Table S3: GO term enrichment analysis of biological processes of transcripts up-regulated in *uis12(-)*.

GO ID	GO term	Total number of genes with this GO term	Number of transcripts up-regulated[a] in *uis12(-)* with this GO term	Fold enrich-ment	Odds ratio[b]	P-value[c]
GO:0070988	demethylation	1	1	25.2	>100	0.04
GO:0006002	fructose 6-phosphate metabolic process	1	1	25.2	>100	0.04
GO:0009107	lipoate biosynthetic process	1	1	25.2	>100	0.04
GO:0009106	lipoate metabolic process	1	1	25.2	>100	0.04
GO:0033615	mitochondrial proton-transporting ATP synthase complex assembly	1	1	25.2	>100	0.04
GO:0000715	nucleotide-excision repair, DNA damage recognition	1	1	25.2	>100	0.04
GO:0008616	queuosine biosynthetic process	1	1	25.2	>100	0.04
GO:0046116	queuosine metabolic process	1	1	25.2	>100	0.04
GO:0034472	snRNA 3'-end processing	1	1	25.2	>100	0.04
GO:0016573	histone acetylation	4	2	12.6	24.6	0.009
GO:0006473	protein acetylation	6	2	8.4	12.3	0.02
GO:0043248	proteasome assembly	8	2	6.3	8.2	0.04
GO:0022613	ribonucleoprotein complex biogenesis	82	10	3.1	3.6	0.001
GO:0034470	ncRNA processing	64	7	2.8	3.1	0.01
GO:0044085	cellular component biogenesis	133	13	2.5	2.8	0.002
GO:0006396	RNA processing	156	14	2.3	2.6	0.003
GO:0071840	cellular component organization or biogenesis	209	17	2.1	2.4	0.003
GO:0016070	RNA metabolic process	283	19	1.7	1.9	0.01
GO:0090304	nucleic acid metabolic process	362	24	1.7	1.9	0.007
GO:0046483	heterocycle metabolic process	475	26	1.4	1.5	0.047
GO:1901360	organic cyclic compound metabolic process	476	26	1.4	1.5	0.048

[a] mean -fold change values of biological replicate 1 and 2 were used for this GO term enrichment analysis

[b] odds ratio statistics was calculated by Fisher's exact test

[c] *P*-values are from Fisher's exact test

Table S4: Oligonucleotides used in this study.

Name	Restriction site	Sequence	Purpose
**Oligonucleotides used for gene deletion constructs and for integration-specific PCR:**			
5' UIS12 for	SacII	TTTCCGCGGGTAAAGCGTTAATTGTAGCG	Knockout *UIS12*
5' UIS12 rev.	NotI	TTTGCGGCCGCGTTTGGGTTTCAACTATAGC	Knockout *UIS12*
3' UIS12 for	HindIII	TTTAAGCTTCCCCATTATCCCCAATATCG	Knockout *UIS12*
3' UIS12 rev	KpnI	TTTGGTACCGTAGGATATGCATACACAC	Knockout *UIS12*
UIS12 test for	-	CATCCTTACATCTATTGCATACC	Integration *UIS12*
UIS12 test rev	-	GCCTCATTTGGGAATTGGGC	Integration *UIS12*
Tg rev	-	CGCATTATATGAGTTCATTTTACACAATCC	Integration pB3D
Tg for	-	CCCGCACGGACGAATCCAGATGG	Integration pB3D
**Oligonucleotides used for WT-specific PCR and/or qPCR:**			
WT UIS12 rev	-	GCAAGGCAAATTTTTCTCTTTT	WT/qPCR *UIS12*
WT UIS12 for	-	CGAAACCAAAACCTCTATTTCC	WT/qPCR *UIS12*
Puf1 rev	-	AACCCGAATTAACAAAACTTGTAGAAGG	qPCR *Puf1*
Puf1 for	-	ATTTGGGTAATTTTCTGAACAACTTATCG	qPCR *Puf1*
MSP1 for	-	TATCGGTAGTAGCAGCTTCTATGGCATC	qPCR *MSP1*
MSP1 rev	-	TATCGGTAGTAGCAGCTTCTATGGCATC	qPCR *MSP1*
HSP70 for	-	AAGAAGCTGAAGCTGTATGCTCTCC	qPCR *HSP70*
HSP70 rev	-	AGTTCATACCTCCTGGCATTCCTCC	qPCR *HSP70*
GFP for	-	GATGGAAGCGTTCAACTAGCAGACC	qPCR *GFP*
GFP rev	-	AGCTGTTACAAACTCAAGAAGGACC	qPCR *GFP*
DOZI for	-	TGTCGCAAACACATCGAAATCGTG	qPCR *DOZI*
DOZI rev	-	ACCCTAAGTGACCATATCTTCCTG	qPCR *DOZI*
UIS1/IK2 for	-	GAAAAGTATAAGAATAAGTTTGTTAGTC	qPCR *UIS1*/*IK2*
UIS1/IK2 rev	-	GATTTATCCTGAACAATATGAATTCC	qPCR *UIS1*/*IK2*
AMA1 for	-	ATTTGGGTTGATGGTTATTG	qPCR *AMA1*
AMA1 rev	-	TCCTTGTCGAAATTTGGTAG	qPCR *AMA1*
P28 for	-	TGAAATGTAAAGCTGCAGAAGAATGC	qPCR *P28*
P28 rev	-	ACTATCACGTAAATAACAAGTAATGC	qPCR *P28*
Act2 for	-	GTATCTCCTGAAGAGCATCCC	qPCR *ActinII*
Act2 rev	-	AGTGTGAGTTACGCCATC	qPCR *ActinII*
MDV for	-	CCCAGTTAATATAGTATTGATGTGTT	qPCR *MDVI*
MDV rev	-	GTTTTAAGCGCCTCTAAATG	qPCR *MDVI*
SET for	-	TATAATTAAAAGACTATATGTAATATCCCCAGTT	qPCR *SET*
SET rev	-	AATTCGAAATAAATTTGCATTTTCTGGAATATTTC	qPCR *SET*

Müller *et al.*, Figure S1

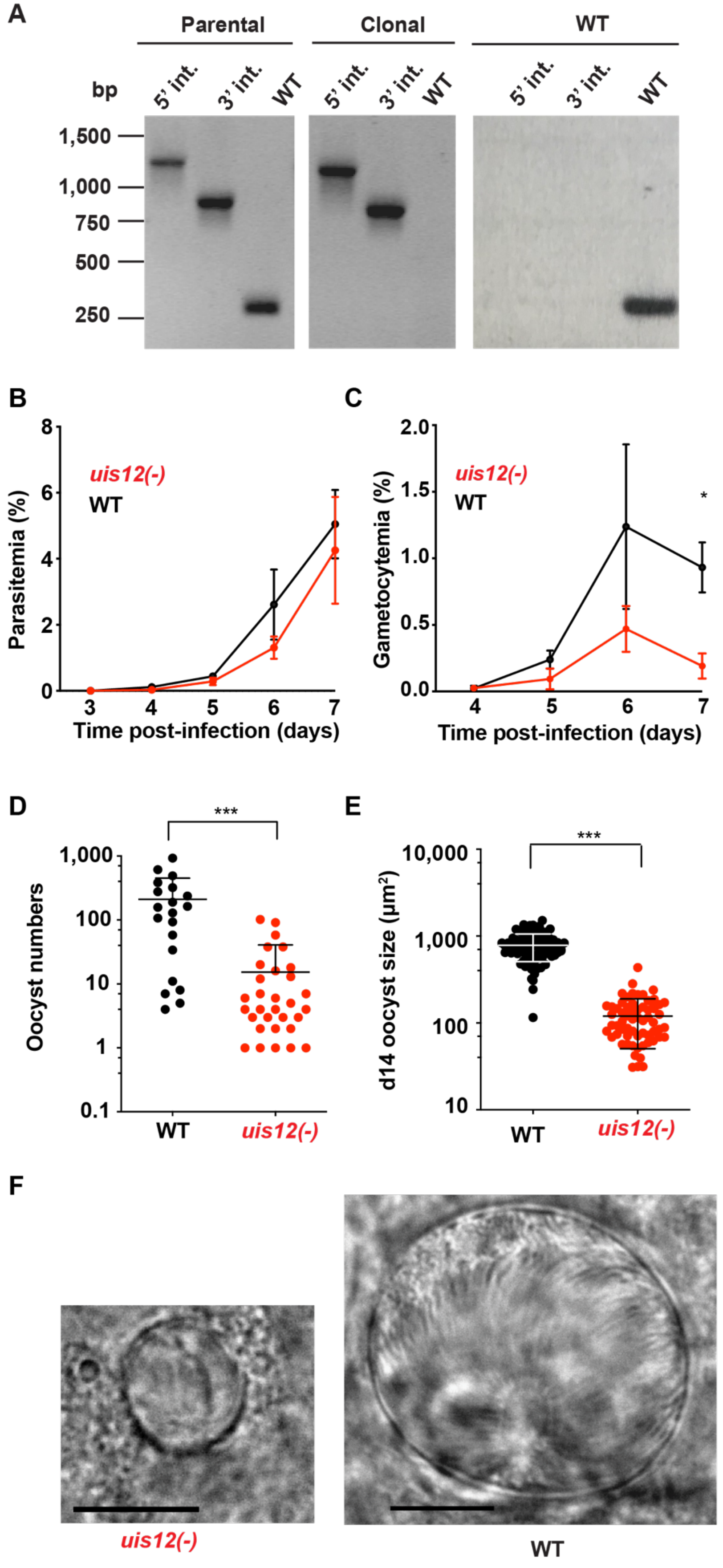

Figure S1: Analysis of a second independent *uis12(-)* clone.

(A) Confirmation of *UIS12* gene disruption by diagnostic PCR. Genomic DNA of parental *uis12(-)*, *uis12(-)* clone 1 and wild-type parasites served as templates. 5'- and 3'-integration-specific primers amplify the predicted fragment only in the recombinant locus. Wild-type-specific primers do not produce a PCR fragment in the recombinant locus and amplified the wild-type locus (right panel). Absence of residual wild-type confirms a clonal *uis12(-)* line (central panel).
(B,C) Time courses of parasitemia (B) and gametocytemia (C) of *uis12(-)* (clone 2, red) and wild-type (WT) (black) parasites, starting three and four days after intravenous injection of 10,000 mixed blood stages into C57BL/6 mice (*n*=5). Daily microscopic analysis of Giemsa-stained blood films was used to determine parasitemia and gametocytemia. Parasitemia and gametocytemia are defined as percentage of asexual parasites and gametocytes per total red blood cells, respectively. Mean values (± S.D.) are shown. *, $p<0.05$ (multiple t-tests, one per row).
(D,E) Oocyst numbers per infected mosquito midgut (D) and d14 oocyst size (μm^2) (E). Data are from two (D) or one (E) independent mosquito infections (*uis12(-)* clone 1, red; WT, black). ***, $p<0.0001$ (Mann-Whitney test).
(F) Shown are representative live micrographs of *uis12(-)* and wild-type oocysts 14 days after an infectious blood meal. *Uis12(-)* clone 1 was used for this experiment. Note the reduced density and size of *uis12(-)* oocysts, and absence of sporozoites in *uis12(-)* oocysts. Magnification, 630-fold; scale bars, 10 µm.

Müller *et al*., Figure S2

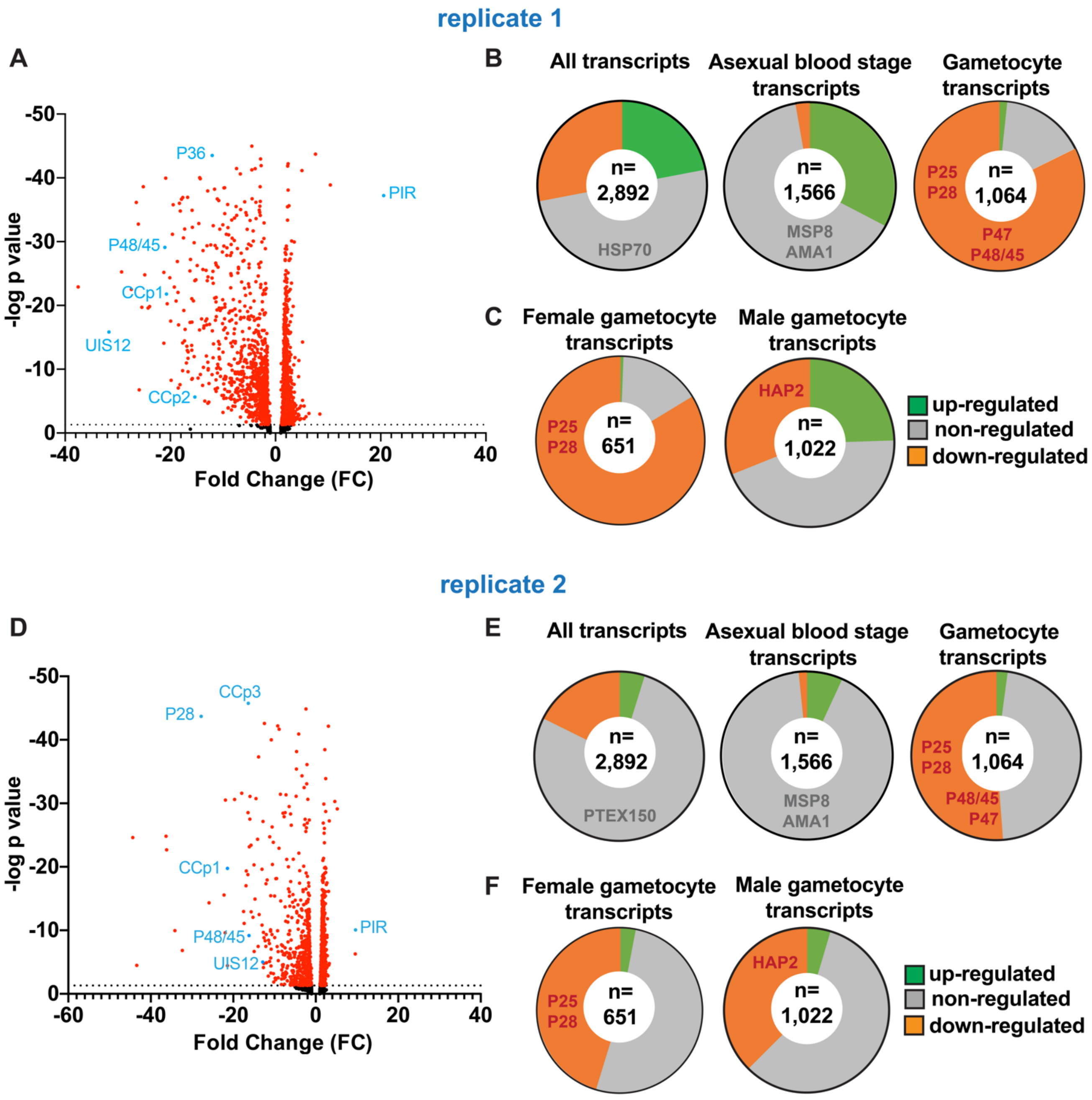

Figure S2: Down-regulation of distinct mRNAs coding for signature gamete-, ookinete- and oocyst-specific proteins in *uis12(-)* blood stage parasites.

Shown are microarray results of total RNA isolated from mixed infected erythrocytes from *uis12(-)* (clone 1)- and WT- infected mice, biological replicate 1 (A-C) and 2 (D-F).

(A,D) Shown is a volcano-plot illustrating the -fold change of the expression levels and the negative log *p*-values of all analyzed 2,890 *P. berghei* genes. The dotted black line represents a *p*-value of 0.05 and all transcripts with a *p*-value <0.05 are shown in red. Exemplary transcripts are highlighted and labeled in blue.

(B,E) Pie charts displaying the proportions of up- (green >2), non- (grey), and down- (orange <-2) regulated transcripts amongst all transcripts (upper left), blood stage-specific transcripts (center) and gametocyte-specific transcripts (upper right) (Otto *et al*., 2014). Exemplary transcripts are listed in the respective region of the chart. The number of transcripts analyzed is shown in a white circle inside the center.
(C,F) Pie charts displaying the proportions of up- (green >2), non- (grey), and down- (orange <-2) regulated transcripts amongst female (left) and male (right) gametocyte-specific transcripts (Yeoh *et al.*, 2017). Exemplary transcripts are listed in the respective region of the chart. The number of transcripts analyzed is shown in a white circle inside the center.

Supplementary References:

Otto, T.D., Bohme, U., Jackson, A.P., Hunt, M., Franke-Fayard, B., Hoeijmakers, W.A., Religa, A.A., Robertson, L., Sanders, M., Ogun, S.A.*, et al.* (2014). A comprehensive evaluation of rodent malaria parasite genomes and gene expression. BMC Biol *12*, 86.

Yeoh, L.M., Goodman, C.D., Mollard, V., McFadden, G.I., and Ralph, S.A. (2017). Comparative transcriptomics of female and male gametocytes in *Plasmodium berghei* and the evolution of sex in alveolates. BMC Genomics *18*, 734.
